# Competitive adsorption of a monoclonal antibody and amphiphilic polymers to the air–water interface

**DOI:** 10.1007/s00249-025-01752-0

**Published:** 2025-05-22

**Authors:** Elise J. Hingst, Michaela Blech, Dariush Hinderberger, Patrick Garidel, Christian Schwieger

**Affiliations:** 1 Institute of Chemistry, Physical Chemistry–Complex Self-Organizing Systems, Martin Luther University Halle-Wittenberg, Von-Danckelmann-Platz 4, 06120 Halle (Saale), Germany; 2 Boehringer Ingelheim Pharma GmbH & Co. KG. Innovation Unit, PDB-TIP, Birkendorfer Str. 65, 88397 Biberach (Riss), Germany

**Keywords:** Poloxamer 188, Polysorbate 20, Infrared reflection–absorption spectroscopy, Surface activity, Monoclonal antibody, Air–water interface, Protein adsorption

## Abstract

**Abstract:**

Understanding the structure and self-organisation of monoclonal antibodies (mAbs) at the air–water interface is crucial for the stability and efficacy of protein drug formulations. This paper investigates the competitive adsorption of mAb and two amphiphilic polymers, poloxamer 188 (P188) and polysorbate 20 (PS20), commonly used to stabilise mAb formulations. Our objective was twofold: to ascertain whether the surfactants in question are capable of preventing mAb adsorption; and to determine whether it is possible to desorb mAb molecules from the air–water interface by surfactant addition. Langmuir film balance measurements and drop shape tensiometry were used to obtain surface pressure and surface tension data. Infrared Reflection–Absorption Spectroscopy (IRRAS) provided information on the surface composition, including the amount of adsorbed molecules. The state adopted by P188 is contingent upon its surface concentration, which determines the self-assembled phases it adopts. We show that the phase state of P188 has a considerable influence on mAb adsorption. The presence of P188 in the brush phase (≥ 0.3 mg/L) consistently inhibits mAb adsorption, but addition of P188 subsequent to the formation of the mAb film does not result in mAb desorption. However, addition of PS20 results in the desorption of freshly-formed interfacial mAb layers of up to two hours’ age, whereas an aged mAb layer of 17 h was unable to be desorbed by PS20. Thus there is a time-dependent reorganisation of mAb at the air–water interface, increasing resistance to desorption, which we discuss in the context of potential intermolecular interactions within the interfacial film.

**Graphical abstract:**

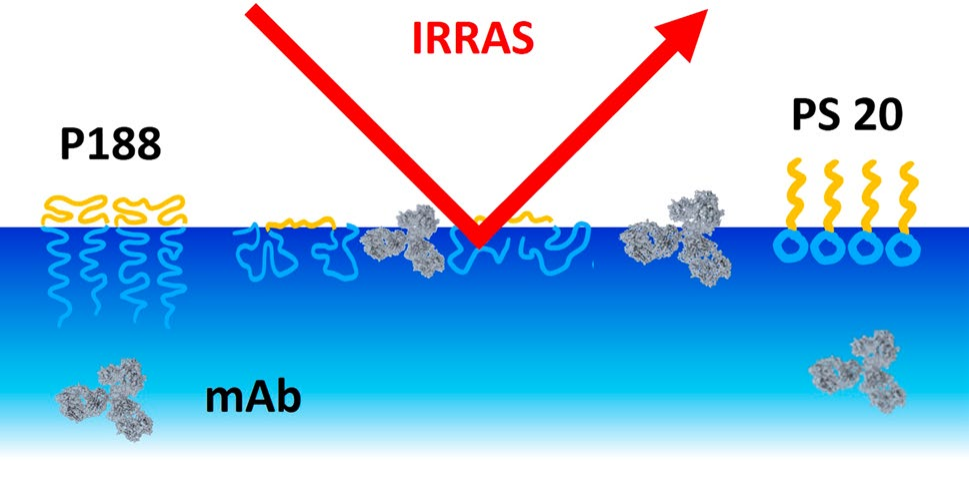

**Supplementary Information:**

The online version contains supplementary material available at 10.1007/s00249-025-01752-0.

## Introduction

Monoclonal antibody (mAb) solutions, particularly at high concentrations, play an important role in the pharmaceutical industry (Garidel et al. 2017, 2009; Chi et al. 2003; Bollenbach et al. 2022; Hollowell et al. 2020; Kamerzell et al. 2011). Subcutaneous injection has become a common method of administering immunoglobulins (Moore and Quinn 2008) and offers a number of advantages compared to intravenous injection, including economy of time (Tjalma et al. 2018), cost saving (Tjalma et al. 2018), and the possibility of simple and independent home therapy (Gardulf et al. 2006). The structure of the extracellular matrix restricts the volume of subcutaneous injection to a maximum of 2 mL (Narasimhan et al. 2012). Consequently, either multiple injections or a highly concentrated product are necessary (Moore and Quinn 2008). The current maximum concentration of liquid monoclonal antibodies (mAbs) used in pharmaceuticals is 150–200 mg/mL (Narasimhan et al. 2012).

The physicochemical properties of highly concentrated mAb solutions present significant challenges in formulation (Chi et al. 2003). On the one hand, there is a tendency towards protein particle formation (Garidel et al. 2015), which can occur at almost every step during mAb production, e.g., manufacturing or storage (Engelsman et al. 2011). Several factors like buffer conditions, temperature, light, and presence of surfaces may foster protein aggregation (Chi et al. 2003). On the other hand, the high viscosities of highly concentrated mAb solutions impede drug formulation development. Hence, syringeability often becomes problematic for subcutaneous injection (Garidel et al. 2017; Rosenberg 2006). Furthermore, the necessity for a longer treatment period and injection pain may occur (Friess et al. 2006; Mathias et al. 2024). Changes in product stability could lead to reduced efficacy and unwanted immunogenic responses, including anaphylaxis (Rosenberg 2006). To impede these reactions and processes and improve formulations, understanding drug stability and mAb behaviour in concentrated solution is of high relevance (Hollowell et al. 2020). Prior research has demonstrated the impact of electroviscous effects (Yadav et al. 2010; Hartl et al. 2017), pH (Garidel et al. 2015; Yadav et al. 2010), buffer conditions (Guo et al. 2012; Kamerzell et al. 2013), and presence of excipients like hydrophobic (Guo et al. 2012) and polar salts (Kamerzell et al. 2013), mono- and disaccharides (Garidel et al. 2015), and amino acids (Schneider et al. 2011) on the viscosity and stability of highly concentrated mAb solutions.

Another important factor is the existence of multiple interfaces (Bee et al. 2011). MAb adsorption to SiO_2_/water interfaces (Pan et al. 2018), which serve as an excellent model for glass/water interfaces, as well as the adsorption to in-line filters used for intravenous administration (Besheer 2017), were observed. Of particular interest is the interaction of mAb with the air–water interface, which is currently the subject of ongoing research (Kannan et al. 2019; Garidel et al. 2021). A key area of investigation is the impact of spontaneous mAb adsorption on the efficacy of protein drug formulations. The processes of protein particle formation and unfolding at the air–water interface remain poorly understood (Bee et al. 2011), and it cannot be excluded that the formulation stability and safety of the formulation may be affected by these processes. It is therefore necessary to develop strategies to impede mAb adsorption to the air–water interface in general. Previous studies have shown that surfactants used as additives or excipients are successful in preventing protein self-assembly at the air–water interface (Bee et al. 2011). Poloxamer 188 (P188) and polysorbate 20 (Tween®20, PS20) are two surface-active excipients approved by the U.S. Food and Drug Administration (FDA) (Research FCfDEa 2023). Polysorbates (PS) are suitable for the use with drugs administered via the main injection routes (subcutaneous, intravenous as well as intramuscular) and represent the primary surfactant added to protein drug formulations (Bollenbach et al. 2022; Gervasi et al. 2018). A number of studies have been conducted with the aim of elucidating the mechanisms underlying the stabilising effect exerted by these excipients in highly concentrated mAb solutions (Garidel et al. 2009, 2021; Kamerzell et al. 2011). Two main paths have been suggested: (i) intermolecular interaction of surfactant molecules with exposed hydrophobic mAb-sites (direct binding) and (ii) surfactant adsorption to the air–water interface with consequential surface shielding for mAb adsorption (Bollenbach et al. 2022; Rabe et al. 2020). Experimental evidence indicates that the enhanced stability of mAb resulting from the addition of PS is primarily attributable to PS blocking the surface, rather than being a consequence of bulk interactions (Garidel et al. 2009). Due to possible enzymatic degradation processes (Wuchner et al. 2022) and its effect on fatty acid particle formation (Glücklich et al. 2020), there is a need for other amphiphilic excipients as alternatives to PS (Dubey and Giovannini 2021). So far, P188 represents an alternative surfactant for parenteral protein drug formulations (Gervasi et al. 2018).

This paper presents the findings of competitive air–water interface adsorption studies involving PS20 and P188 in the presence of mAb. The adsorption kinetics were studied using Langmuir film balance measurements and drop shape tensiometry. Infrared reflection–absorption spectroscopy (IRRAS), was used to derive information on the molecular composition of the mAb/surfactant interfacial films with various surfactant concentrations and film formation procedures (Rabe et al. 2020). The conditions under which mAb adsorption to the air–water interface can be prevented or already formed mAb films can be removed from the interface have been identified.

## Materials and methods

### Materials

**Scheme 1:**
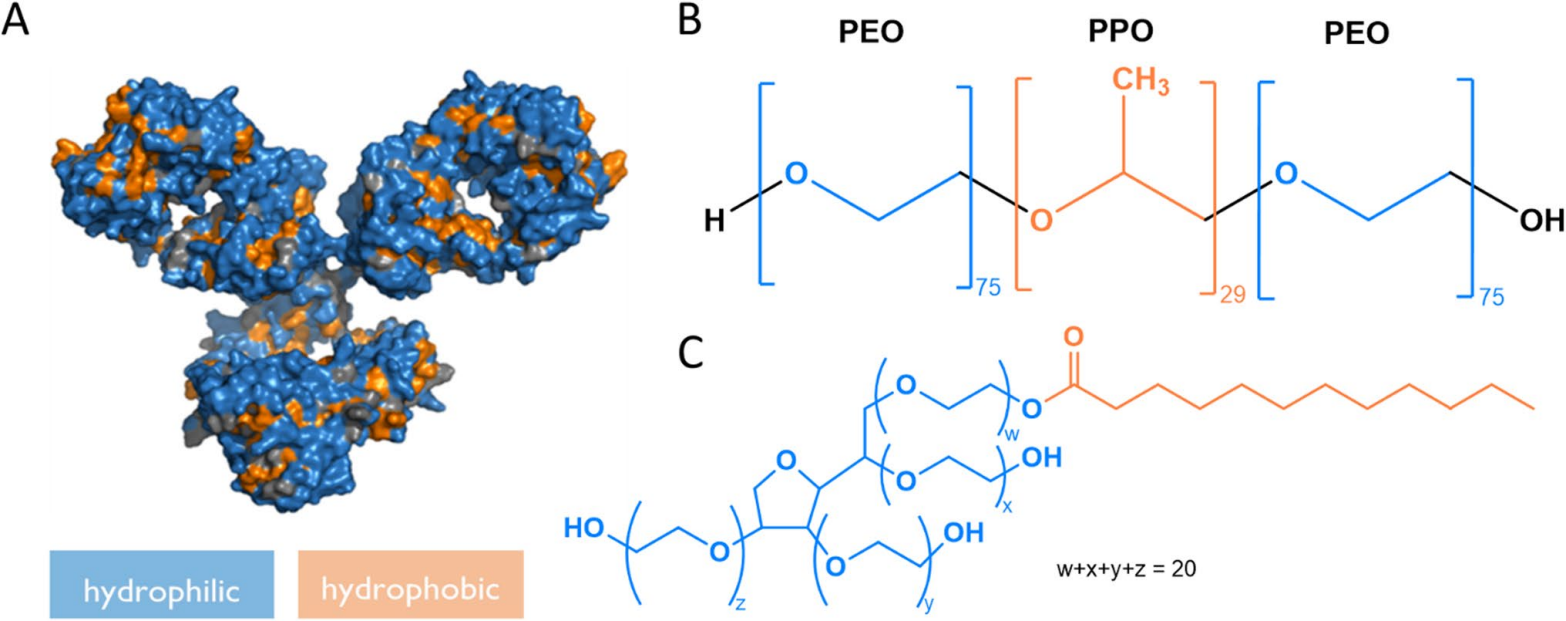
(Chemical) structures of tested substances with color-coded hydrophilic (blue) and hydrophobic (orange) moieties. **A** An example of an antibody structure (pdb: 1IGY), **B** The triblock copolymer P188, *PEO* Poly(ethylenoxide), *PPO* Poly(propylenoxide). **C** Structure of poly(ethylenoxide) sorbitan lauric acid ester, PS20 (color figure online)

The structures of the employed substances are shown in Scheme 1. ChemDraw 22.2.0 and PyMOL 2.3.3 were used as graphic software. The monoclonal antibody (mAb) used in this study was provided by Boehringer Ingelheim Pharma GmbH & Co. KG (Biberach, Germany). It belongs to the IgG1 antibody class. The structure is analogous to, though not identical to, that depicted in Scheme 1A. The average molar mass of the used mAb is 146,000 g/mol. Poloxamer 188 (P188, Scheme 1B) and polysorbate 20 (PS20, high purity qualities, Scheme 1C) were obtained from BASF and Croda International, respectively. P188 has an average molecular weight of 8500 g/mol. The average molecular weight used for PS20 is 1228 g/mol. Chloroform, ethanol and methanol (all HP grade) were obtained from Carl Roth GmbH & Co.KG (Karlsruhe, Germany). L-Histidine and L-Histidine ∙ HCl ∙ H_2_O were obtained from Ajinomoto OmniChem (N.V., USA). Ultra-pure Milli-Q water (κ < 0.055 µS/cm) was used for all preparations and experiments.

### Sample preparation

In order to prepare the protein and surfactant solution, the required amount of components were weighed using an analytical balance and subsequently dissolved in 24.4 mM histidine buffer (11.4 mM L-Histidine, 12.8 mM L-Histidine HCl H_2_O, pH = 6.0 ± 0.2). This buffer composition is used for all preparations and experiments described in this paper.

P188 solutions were dissolved in a concentration range from 0.5 mg/L (approximately 0.06 µM) to 20 g/L (approximately 2.4 mM) in histidine buffer and mixed on a vortex mixer. For the measurement of compression isotherms, a 0.122 mM solution was prepared by dissolving P188 in chloroform.

PS20 solutions were prepared by dissolving PS20 in a concentration range from 1.2 mg/L (approximately 0.001 mM) to 12.28 g/L (approximately 10 mM) in histidine buffer. The solutions were mixed by gentle agitation to prevent strong foaming.

MAb solutions were diluted in a concentration range from 50 mg/L (approximately 0.3 µM) to 20 g/L (approximately 0.1 mM) in histidine buffer and mixed by gentle agitation. The stock solution had a concentration of 90 g/L.

### Monolayer preparation

Distinct volumes of proteins and surfactants dissolved in 24.4 mM histidine buffer (pH = 6.0 ± 0.2) were carefully injected into the histidine buffer subphase of the thoroughly cleaned and filled film balance trough using glass syringes (Hamilton Bonaduz, Bonaduz, Switzerland). All measurements were performed at 20 °C.

### Langmuir film balance measurements at the air–water interface

All Langmuir film balance measurements were performed on two home-built circular PTFE (polytetrafluoroethylene)-troughs (Scheme S1) with a volume of about 10 mL. A perspex sheath protects the surface from draught and dust. Four cellulose- and water-filled bowls under the sheath ensured a constant protein concentration in the subphase by securing constant air humidity and therefore preventing subphase-evaporation. The surface pressure was measured by a microbalance (Riegler und Kierstein GmbH, Berlin). A filter paper (Wilhelmy plate) was used as surface pressure probe. All measurements were performed at 20 °C ± 0.1 °C, regulated by a water bath (Thermostat F6, Haake, Karlsruhe, Germany).

Before and after each measurement the troughs were cleaned with Hellmanex™ (Hellma GmbH & Co. KG, Müllheim) in a 1:100 dilution for at least 20 min exposure time and subsequently with ultra-pure water. A fresh Wilhelmy plate was attached after every experiment involving protein. A clean trough is characterized by a rupture of the water layer without droplet formation when aspirating the solution.

The surface tension σ of ultra-pure water (72.8 mN∙m^−1^) (Vargaftik et al. 1983) and the surface tension of air (0 mN∙m^−1^) at 20 °C were used as references for calibrating the pressure sensor. First, water was filled into the trough and the device-specific fill level was adjusted using a height-standardised cannula. Adjusting the specific fill levels (± 0.5 mm) for each device is necessary to obtain optimal, reproducible results. Subsequently, the surface pressure π was regulated to the value of 0 mN∙m^−1^. Calibration at the second point was performed by suctioning the subphase until the Wilhelmy plate was no longer covered, where π was set to 72.8 mN∙m^−1^. Note that the surface pressure is calculated as $\pi = \sigma_{{H_{2} O}} - \sigma$.

Swift operation is important as otherwise, the calibration value would be distorted by evaporation effects. Once the calibration process was complete, the histidine buffer was introduced to the trough, and the temperature was allowed to stabilise before adjusting the device-specific filling height once more. Following calibration, the troughs filled with histidine buffer were allowed to reach equilibrium for a period of 20 min.

The sample solution was injected through a lateral hole in the trough or through a lockable aperture in the sheath, depending on the injected component. PS20 was injected through the sheath to prevent trough-leakage and consequential adulteration of the subphase fill level. During the measurement, the subphase was gently stirred by a magnetic stirrer.

### Infrared reflection-absorption spectroscopy (IRRAS) at the air–water interface

A scheme of the instrument is shown in the supplementary information (Scheme S2). IRRAS measurements were performed on a BRUKER Vector 70 FT-IR spectrometer including an A511 reflection unit (Bruker Optics, Ettlingen, Germany) and a liquid nitrogen cooled MCT detector. The Langmuir trough setup (Riegler & Kierstein, Potsdam, Germany) contains a circular sample trough with a diameter of 6 cm, equipped with a Wilhelmy plate as pressure probe, and a reference trough (30 × 6 cm^2^). In both troughs the temperature was held constant at 20 °C by a thermostat (F6, Haake, Karlsruhe, Germany). Using a shuttle-system, either trough can be brought into the focus of the IR beam as needed. Defined and constant fill levels in the troughs are important to achieve reproducible results. This is ensured by a computer-controlled pumping unit connected to a laser reflection signal which provides the fill level data. To enable a stable atmosphere a perspex sheath was placed over the Langmuir trough setup and the IR-reflection unit (Schwieger et al. 2012).

Both troughs were filled with the same buffer subphase and the filling level was adjusted as described above. Prior to sample injection, up to ten buffer–buffer spectra were recorded to check the purity of the trough and the surface. After that, 5 µL of the sample solution were gently injected into the subphase at two different positions and in the lane of the magnetic stirrer.

The angle of incidence of the IR-beam can be varied between 25° and 70°. The polarization of the IR-beam can be varied between perpendicular (s) or parallel (p), with respect to the plane of incidence. Time dependent series of spectra were obtained in steps of 5-min-intervals between two sample measurements. All adsorption measurements were conducted with an s-polarized IR-beam, an incidence angle of 40°, a scan number of 1000 scans per spectrum, a resolution of 8 cm^−1^ and a zero-filling factor of 4. The penetration depth of the IR-beam is 0.5–1.0 µm. For recording and initial analysis the spectroscopy software OPUS (Bruker Optics) was used. Isotherms were analysed and plotted with the software OriginPro 2019.

### Spectral simulations and fits

First, an offset was subtracted from the measured spectra such that the wavelength region between 2500 cm^−1^ and 2600 cm^−1^ was set to an average intensity of zero. This was followed by subtraction of averaged spectra of the pure buffer to compensate for atmospheric influences. For best compensation of atmospheric and buffer influences, the averaged buffer spectrum was scaled such that after subtraction the variance of the second derivative was minimal. Since the H_2_O bending vibration (1650 cm^−1^) partially overlaps with the amide I band at 1658 cm^−1^, a computer simulated spectrum of a non-adsorbing layer with a thickness of 1 nm and pure water as a subphase with its optical parameter taken from Bertie et al. (1996) was subtracted (Vargaftik et al. 1983). The simulated spectrum was scaled such that after subtraction the total intensities were minimal in the range of 3680 cm^−1^ to 3450 cm^−1^. From the scaling of the simulated spectrum, we were able to estimate absolute values of the layer thickness of adsorbed layers in nm. Eventually, characteristic bands of the compounds present at the interface (Table S1) were integrated to obtain a measure for their interfacial concentration. The integral areas are given in milli-reflectance-absorbance per centimeter (mRA/cm).

### Drop shape tensiometry

The drop profile analysis tensiometer PAT-1 M by SINTERFACE Technologies (Potsdam, Germany) in the pendant drop mode was used for adsorption experiments. Prior to every experiment, tubes were rinsed with ultra-pure water. To examine adequate purity, the surface tension of a water drop (*V* = 35 µL) was measured. The surface tension must stay constant in a range of 71.0–73.0 mN∙m^−1^ for at least 5 minutes. If this was not the case, the system was rinsed with a 1 % Hellmanex™ solution and with ultra-pure water again. Remaining water was rinsed off the tube and the sample was aspirated. The first two to three drops of the sample solution were discarded. All measurements were performed with a cannula of 3 mm diameter and a drop volume that was held constant at 20 µL. After finishing the experiment, the system was rinsed with ultra-pure water. The software PAT-1 M was used. Data points were saved every second, which includes values of surface tension (mN∙m^−1^), drop area (mm^2^), drop volume (mm^3^) and profile error. For calibrating the device, camera calibration was performed using a spherical stainless-steel probe (*d* = 3 mm). The focus can be adjusted in the setup of the program as well as with a setting wheel at the device.

## Results and discussion

### Surface activity of pure compounds

Before addressing the surfactant-mAb interactions at the air–water interface, the pure components were measured separately for proper referencing. We used a combination of drop shape tensiometry and Langmuir film balance measurements to study the surface activities of the components as a function of their bulk concentration. Both methods are complementary and are used to obtain cross-validated results.

Drop shape tensiometry (pendant drop) needs much lower sample volumes (about 200 µL) than Langmuir film balance measurements (up to 10 mL). Furthermore, drop shape tensiometry enables experiments with high bulk concentrations (> 20 g/L) and long-time measurements extending to several days (with adaptive drop volume or drop area control via computer and camera). Both types of experiments cannot be realised with the film balance. On the contrary, film balance measurements are more sensitive and can be used for very low concentrations (< 1 mg/L). Since very small changes in surface tension resulting from low volume concentrations do not lead to detectable variations of the drop shape, drop shape tensiometry is not suitable for measurements below 1 mg/L.

### P188 behaviour at the air–water interface

**Scheme 2:**
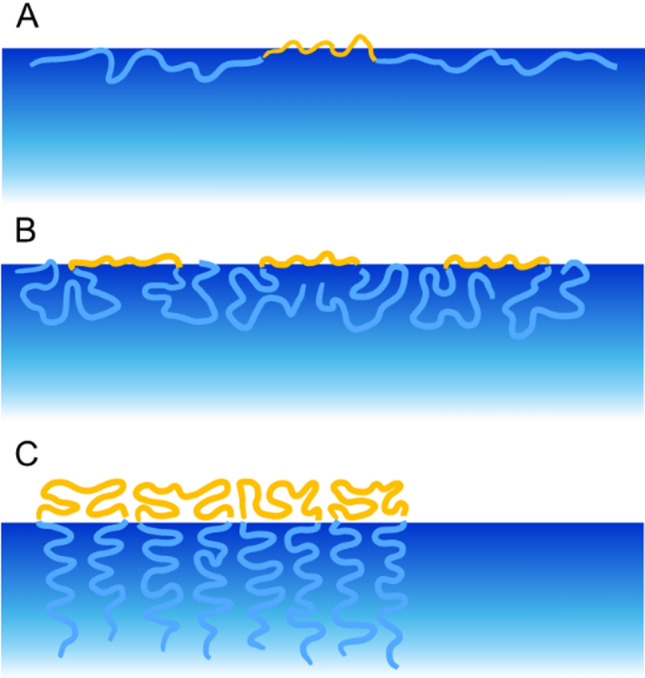
Schematic representation of the behaviour of P188 at the air–water interface with increasing surface pressure adapted from Haefele et al. (2006). At low surface pressures, the surfactants lie flat on the surface, while at higher pressures, the PEO blocks protrude into the bulk phase. **A** “pancake” phase, **B** “mushroom” phase, **C** “brush” phase. The hydrophobic PPO blocks are orange-coloured, while the hydrophilic PEO blocks are blue-coloured (color figure online)

**Fig. 1:**
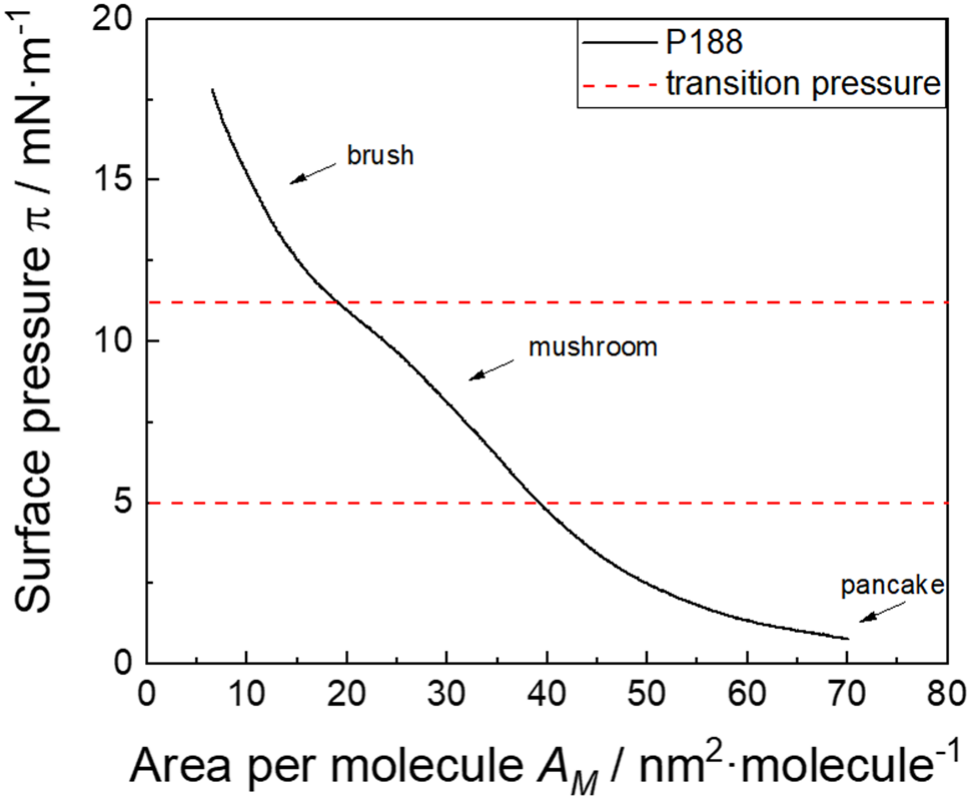
Compression isotherm of P188, performed on histidine buffer (pH = 6.0 ± 0.2) at a temperature of T = 20 °C. The dotted red lines show the transition pressure from “pancake” to “mushroom” at π = 5 mN∙m^−1^ and from “mushroom” to “brush” phase at π = 11 mN∙m^−1^ (color figure online)

P188 exists in different self-assembled phases at surfaces, depending on its surface concentration (Gennes 1987; Muñoz et al. 2000), which are shown in Scheme 2. Injecting P188 molecules into water leads to adsorption of the molecules the air–water interface due to the hydrophobic PPO-blocks (orange coloured in Scheme 2). Since the inherently hydrophilic PEO blocks (blue coloured) also possess a certain degree of hydrophobicity, they also accumulate at the air–water interface at low surface concentrations, forming flat structures, referred to as “pancakes” (Gennes 1987). Increasing surface concentration reduces the available space, causing the EO units to be displaced from the surface into the subphase. Thereupon, mushroom shaped structures (“mushroom” phase) form, followed by brush-like structures (“brush” phase). These phase states have been described previously in general for amphiphilic block copolymers by Haefele et al. (2006). Furthermore, Haefele et al. (2006) describe a film organization state called “cigar”, which follows the “brush” state. With our methods we were not able to detect a higher condensed state than “brush”, because no further plateau above π = 11 mN∙m^−1^ is clearly discernible in the compression isotherm (see Fig. 1). Consequently, we refer to the highly condensed phase as the “brush” phase hereinafter. A compression isotherm of P188 is shown in Fig. 1, which relates to previous experiments (Muñoz et al. 2000; Powroznik 2019). The “mushroom”- “brush” transition can be observed in the compression isotherm as a pseudo-plateau at 11 mN∙m^−1^, whereas the “pancake”- “mushroom” transition is gradual. Herein, we refer to P188 films between 5 and 11 mN∙m^−1^ as P188 “mushroom” phase.

### Adsorption behaviour

**Fig. 2:**
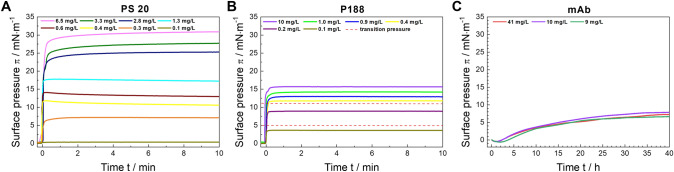
**A** Adsorption isotherms of polysorbate 20 (PS20), poloxamer P188 and a monoclonal antibody (mAb) at different subphase concentrations determined by Langmuir film balance method. Measurements were performed on histidine buffer (pH = 6.0 ± 0.2) at a temperature of *T* = 20 °C. Note the identical surface pressure axes scales while the time axes are different. **B** The upper red dashed line shows the transition pressure of the “mushroom” phase to the “brush” phase at π = 11 mN∙m^−1^, obtained from the compression isotherm in Fig. 1. The lower red dashed line shows the transition pressure of the “pancake” phase to the “mushroom” phase at about π = 5 mN∙m.^−1^ (color figure online)

The adsorption isotherms (Fig. 2) show quite different adsorption kinetics for the three studied components when they are injected into the aqueous subphase. Both P188 and PS20 exhibit greater surface activity and a faster adsorption rate at the interface than the mAb. Within 2 min, a stable surface pressure π is achieved. P188 reaches the saturation equilibrium pressure at 18 mN∙m^−1 ^(Powroznik 2019) and PS20 at 35 mN∙m^−1 ^(Rabe et al. 2020), indicating a higher surface activity of PS20 when compared to P188.

The surface behaviour of the mAb is shown in Fig. 2C. The difference in adsorption kinetics compared to the surfactants is striking. MAb adsorption proceeds slower, i.e., the equilibration takes several hours and the mAb surface activity (ca. 7 mN∙m^−1^) is clearly lower than that of the surfactants. This can be explained by the much lower amphiphilic character of the mAb. While P188 and PS20 have clearly discernible hydrophilic and hydrophobic moieties, hydrophobic areas of the mAb are distributed throughout the large protein molecule and partially hidden inside the structure. These hidden areas may be exposed at the surface in case of adsorption which would be one reason for the significant differences in kinetics and activity as compared to the surfactants (Koepf et al. 2017; Kanthe et al. 2021). The slow adsorption kinetic of the mAb can in addition be explained by its high molecular weight of about 146,000 g/mol. Compared to P188 (8,500 g/mol) and PS20 (1,228 g/mol), the mAb is a large molecule which diffuses much slower to the air–water interface. Molecular dynamic simulations yielded a radius of gyration of 4.1 ± 0.87 nm per P188 molecule was obtained (Adhikari et al. 2016). The radius of gyration of a PS20 micelle was determined to be 2.1–2.2 nm by molecular dynamic simulations (Lapelosa et al. 2016) which aligns well with experimental results obtained by dynamic light scattering (2.4 nm) (Carnero Ruiz et al. 2003). MAb gyration radii values are at about 5 nm, calculated by Guinier analysis (Dear et al. 2019).

In addition, we performed drop shape tensiometry measurements at similar conditions (Figure S1). The results are in accordance with those obtained by the film balance measurements described above.

The generated data clearly demonstrate that the investigated surfactants exhibit accelerated adsorption kinetics and enhanced surface activity. Consequently, these surfactants should be capable of protecting the surface and preventing mAb adsorption, either when administered prior to or when administered simultaneously with the mAb. This is, however, only true when no synergistic effects and no interactions between mAb and the surfactants are assumed. In the following paragraphs the competitive adsorption of mAb and P188 as well as mAb and PS20 is investigated and discussed in detail.

### Competitive adsorption of monoclonal antibody and surfactants at the air–water interface

Competitive surface adsorption experiments were performed to study how mAb behaves in presence of either P188 or PS20. To this end, injection sequence, time of injection and concentrations were varied. The experiments were conducted on an adsorption Langmuir film balance. Furthermore, infrared reflection–absorption spectroscopy (IRRAS) was performed to obtain information on the molecular surface composition, which is possible since the different adsorped molecules lead to distinguished bands in the infrared reflection-absorption spectra.

All experiments were performed with a subphase composed of 24.4 mM histidine buffer (pH = 6.0 ± 0.2). The experiments were conducted with concentrations below the critical micelle concentration (*cmc*) of 0.48 mM for P188 (Merck and Deutschland 2023; Chunhachaichana and Srichana 2019) and 0.059 mM for PS20 (Knoch et al. 2021). Plots for calculating the *cmc* can be found in the Supplementary Information (Figure S2).

**Scheme 3:**
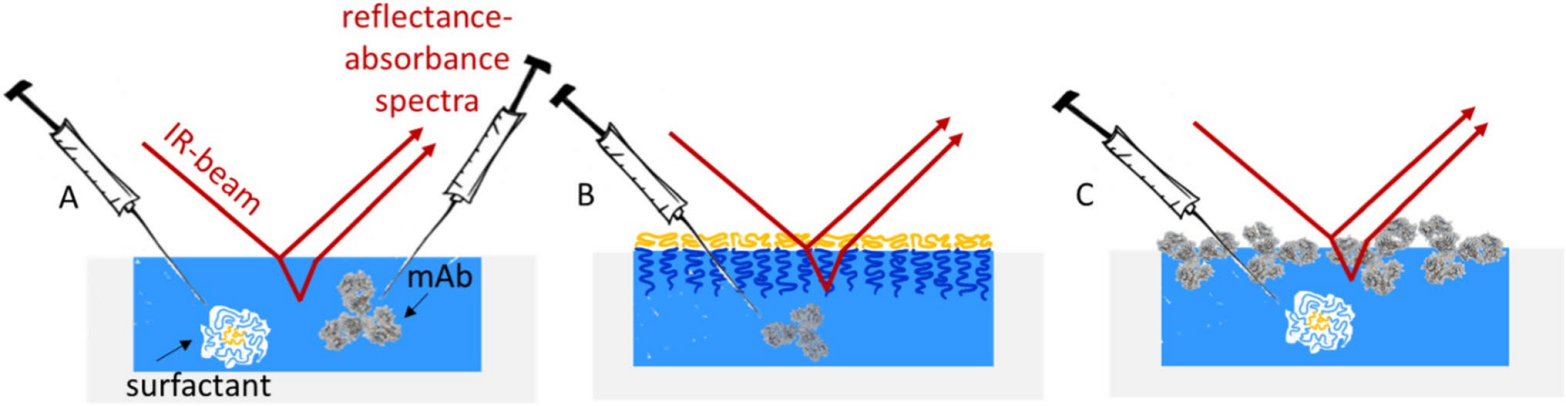
Used experimental setups. Surfactant constitutes either P188 or PS20. **A** Simultaneous injection of surfactant and mAb into the pure buffer (24.4 mM histidine, pH = 6.0 ± 0.2), **B** Injection of mAb underneath a pre-formed surfactant layer, **C** Injection of surfactant underneath a pre-formed mAb layer

In the following we describe three different sets of experiments as schematically shown in Scheme 3. That is (A) simultaneous injection of either surfactant and the mAb into the subphase (B) injection of mAb underneath a pre-formed film of surfactant, and (C) injection of the surfactant underneath a pre-equilibrated film of mAb. Thereby case A) simulates the situation where a surfactant and mAb are both present in solution and a new surface is created by shaking or handling a mAb formulation.

**Fig. 3:**
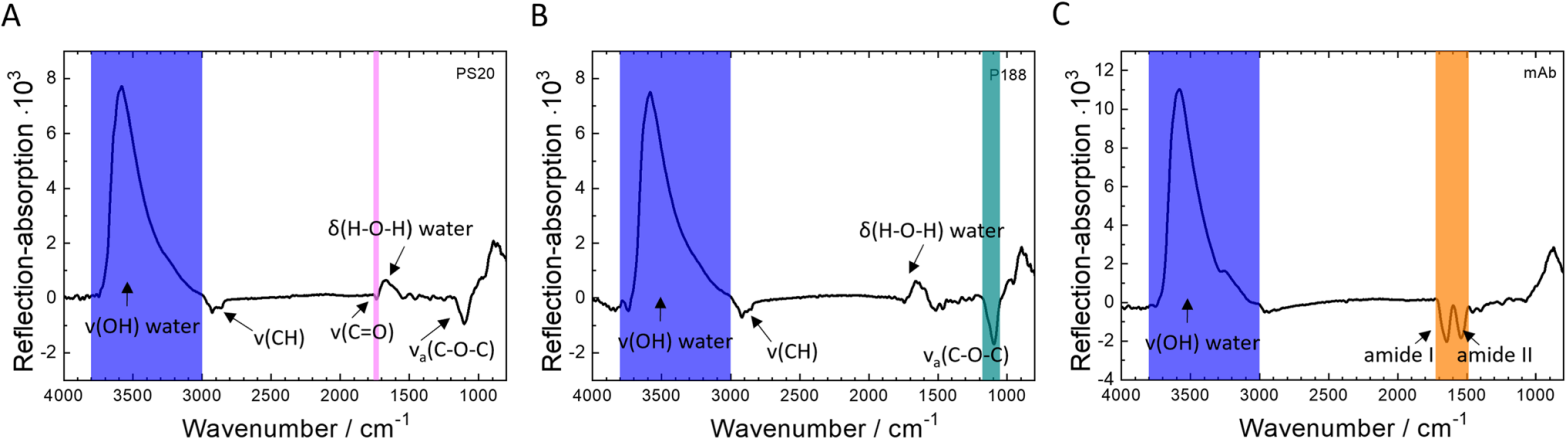
Infrared reflection–absorption (IRRA) spectra of the pure compounds at the air–water interface. **A** PS20 after 30 min of adsorption, subphase concentration 1 mg/L, **B** PS20 after 30 min of adsorption, subphase concentration 1 mg/L, **C** after 15 h of adsorption, subphase concentration 5 mg/L. ν, stretching vibration; δ, deformation vibration. Integration limits of the respective bands are shown as coloured, transparent boxes (color figure online)

In Fig. 3, IRRA spectra of the pure components at the air–water interface are shown for reference. For each compound, unique characteristic reflection–absorption bands, that do not overlap with absorptions of the other compounds were chosen as reporter bands. Their integrals are used as measures of the amount of adsorbed component at the air–water interface. For PS20 that is the carbonyl stretching vibration ν(C = O), for P188 the asymmetrical ether stretching vibration ν(C–O–C) and for the mAb the amide I and amide II band. The water stretching vibration ν(HOH) originates from absorption of the aqueous subphase and is used for the determination of the layer thickness as described in Schwieger et al. (2012). Before further evaluation, the subphase water contribution is excluded from the spectra by subtraction of simulated IRRA spectra of a non-absorbing layer with adjusted layer thickness. The resulting water-compensated spectra are shown in Figure S3.

The concentrations of P188, PS20 and mAb that are the subject of this study are well below those typically used in pharmaceutical biologics, with the concentration of mAb being 18 times lower. However, the selected molar ratios of surfactant to mAb are consistent with those found in commercially available biological drugs. Based on the area requirement calculations for mAb (SI), we estimated that the air–water interface is almost entirely covered by mAb molecules in the selected experimental conditions. Therefore, higher bulk concentrations are not expected to lead to higher surface concentrations or different interactions with the surfactants at the interface. It is assumed that the described adsorption behaviours and interactions between mAb and surfactants at the air–water interface are representative of those occurring in solutions of higher concentration.

### Simultaneous injection of surfactants and antibody

**Fig. 4:**
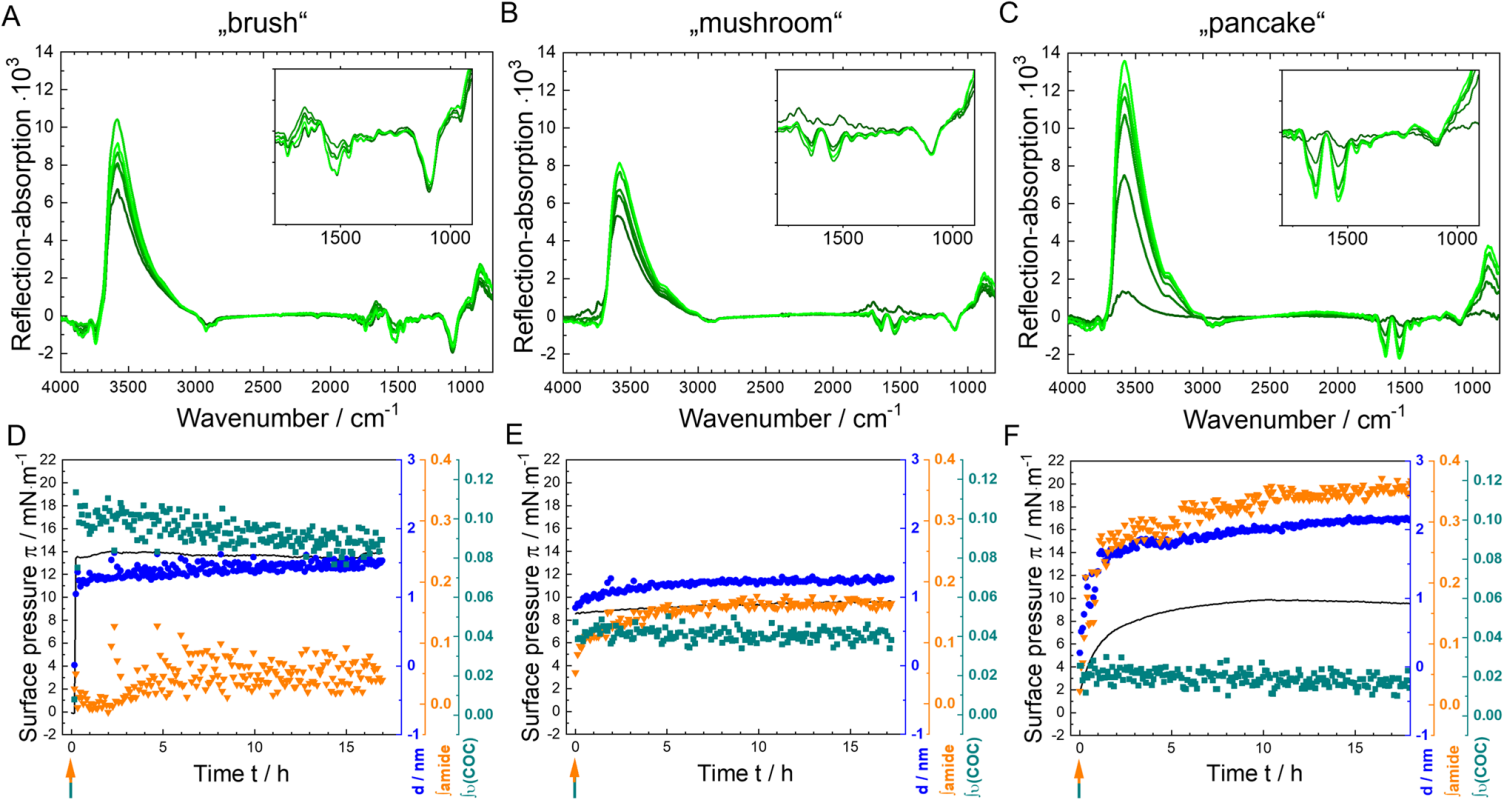
Competitive surface activity experiments P188 and mAb, using simultaneous Infrared reflection–absorption spectroscopy (IRRAS) and surface tension measurements. **A** simultaneous injection of mAb (*c*_sub_ = 5 mg/L) and P188 in different concentrations (**A**, **D**: “brush” 1 mg/L, B,E: “mushroom” 0.13 mg/L, C,F: “pancake” 0.11 mg/L) was performed. Measurements were performed on 24.4 mM histidine buffer (pH = 6.0 ± 0.2) at a temperature of *T* = 20 °C. **A**–**C** IRRA spectra recorded 5 min to 15.5 h after mAb and P188 injection. The insets show an enlarged part of the spectra in a wavenumber range of 1800 cm^−1^ to 900 cm^−1^. The colour representation corresponds to different adsorption times. Darker shades of green indicate earlier spectra, while lighter shades of green indicate later spectra. **D**–**F** Surface pressure π (mN∙m^−1^) (black line), layer thickness *d* (blue circles ●), integral of amide I and amide II bands (green triangles ▼) and integral of asymmetrical C–O–C-stretching vibrational band centred at 1100 cm^−1^ (olive squares ■) as a function of time (h). The green-orange coloured arrow shows the injection time of P188 and mAb into the subphase (color figure online)

To study the direct competitive behaviour of the components, IRRA spectroscopic measurements with simultaneous injection of both components, mAb and either P188 or PS20 at final bulk ratios of  ≥ 1 or 2.6 surfactant molecules per antibody molecule, were performed. A mAb subphase concentration of 5 mg/L was used for each measurement. IRRA spectra and time dependent integral intensities of the marker band for the experiments with P188 and mAb are shown in Fig. 4. In this experimental setup, a P188-phase dependent behaviour becomes evident.

When working with bulk concentrations that lead to P188 self-assembly in the “brush” phase, only P188 adsorbs to the air–water interface and mAb is not able to co-adsorb, which can be seen in Fig. 4A and D. Figure 4A shows the IRRA spectra of the measured P188-mAb-mixture. Figure 4D shows the time dependent evolvement of surface pressure π (black line), the layer thickness *d* (blue dots), the integral of amide-I and amide-II-bands of the mAb (orange triangles), and the integral of the asymmetrical C–O–C-stretching vibration of P188 centred at 1100 cm^−1^ (green squares). The surface pressure increases immediately after the simultaneous injecting of P188 and mAb, reaches its maximum and remains constant over the time of the measurement. The fast increase in surface pressure as well as its equilibrium value of ca 15 mN∙m^−1^ is particularly attributable to the adsorption of P188, as evidenced by the results obtained from the pure component measurements described above. The constant surface pressure indicates a fast, unfluctuating surface layer formation. The average layer thickness is determined from the ν(HOH) vibration of the subphase water to be approximately 1.5 nm and fits reasonably well with data published in previous studies (Ortega-Vinuesa et al. 1998). Based on the amide and ν(C–O–C) band integrals, the average surface layer composition can be estimated. The ν(C–O–C) integral increases together with the surface pressure immediately with the injection to ca. 0.1 mRA/cm, indicating fast and stable adsorption of P188 to the interface. In contrast, the amide band integral remains at a negligible level (zero or close to zero) throughout the course of the experiment, indicating that the mAb is unable to adsorb to the interface at these conditions. Correspondingly, in the associated IRRA spectra (Fig. 4A) the characteristic ν(C–O–C) vibration of P188 at 1100 cm^−1^ is strongly pronounced, whereas in the wavenumber range of the amide I and II bands from 1725 cm^−1^ to 1485 cm^−1^, only the deformation vibration of water δ(HOH) at 1650 cm^−1^ is discernible.

In the presence of P188 at concentrations that result in the formation of the “mushroom” phase, the time-dependent integrals of the amide bands and the ν(C–O–C) band (Fig. 4E) demonstrate that P188 molecules exhibit a rapid adsorption rate at the air–water interface, albeit to a lesser extent than that observed for P188 in “brush” phase concentration (0.04 mRA/cm). Correspondingly, the surface pressure increases only to π ~ 8 mN∙m^−1^ and the average layer thickness stabilizes at ~ 1.2 nm. However, the reduced P188 adsorption capacity enables the parallel adsorption of mAb at the interface, as evidenced by the gradual and slow increase in the amide band integral to approximately 0.18 mRA/cm. Qualitatively, the changes in layer composition can be substantiated through an examination  of the IRRA spectra (Fig. 4B). There is a discernible reduction in the P188 characteristic ν(C–O–C) vibration at 1100 cm^−1^, while the deformation vibration of water δ(H–O–H) at 1650 cm^−1^ regresses and the amide bands at 1725 cm^−1^ to 1485 cm^−1^ emerge with greater clarity.

When the mAb is injected together with P188 in concentration that allow only the formation of a “pancake” phase (Fig. 4C and F), barely any P188 molecule adsorbs to the surface allowing maximal adsorption of the mAb. Thus, the P188 characteristic ν(C–O–C) vibration is only marginally discernible in the spectra, while the amide bands centred at 1550 cm^−1^ and 1650 cm^−1^ are clearly visible (Fig. 4C). Figure 4F shows amide band integrals having reached the maximum value of this series of measurements (0.35 mRA/cm), while the integral values in the ν(C–O–C) region remain close to zero. Despite the presence of P188 in the solution, mAb is able to adsorb to the air–water interface. This is a competitive process with slower increase in surface pressure and layer thickness, compared to the cases where the adsorption is dominated by P188. Interestingly, the final average layer thickness is higher (2 nm) than that of a P188 brush (1.5 nm) and that of the mixed P188/mAb layer (1.3 nm).

Notwithstanding the distinct differences in these three measurements, it remains uncertain whether the observed behaviour is inherently linked to the phase state of P188 or merely a consequence of a concentration dependence. In a pharmaceutical product, the highly condensed phase (“brush”) of P188 is predominant when the three concentrations are considered.

The results of the experiments conducted on the simultaneous injection of mAb and PS20 are presented in the supplementary information (Fig. S4). The results are comparable to those obtained with “brush” phase P188, i.e., exhibiting an almost instantaneous increase in surface pressure, layer thickness, and the integral of the PS20 marker band, with a near-zero integral of the amide bands. This shows that PS20 is also capable of rapidly adsorbing to free surfaces, and thereby preventing mAb adsorption. PS20 provides complete coverage of the surface, thereby preventing mAb adsorption at all concentrations studied.

### Antibody injection after formation of a surfactant film

In this series of experiments, the surfactant films were formed prior to the mAb injection. Consequently, mAb can adsorb to defects in the layers, replace the surfactants from the interface, or mAb can adsorb to the surfactant layers. These mechanisms can be investigated by stepwise injection of (i) the surfactents and (ii) the mAb. Because in this experimental design we can determine starting values of surface pressure, layer thickness and band intensities, we can attribute any changes after mAb injection to film reorganisation.

As in the initial set of experiments, P188 concentrations were chosen, such that P188 forms different self-assembled phases at the air–water interface. This allows to investigate whether the poloxamer layer structure affects the interaction between the mAb and the surfactant. Also, PS20 was tested in various subphase concentrations in Langmuir film balance measurements. The results are shown in the supplementary information (Fig. S5). From these experiments it can be deduced that mAb is able to insert into films formed by P188 at concentrations *c*_P188_ < 1 mg/L and films formed by PS20 concentrations *c*_PS20_ < 0.11 mg/L. This shows that PS20 has a higher propensity to shield the interface, i.e., lower amounts of PS20 are necessary to prevent mAb adsorption.

**Fig. 5:**
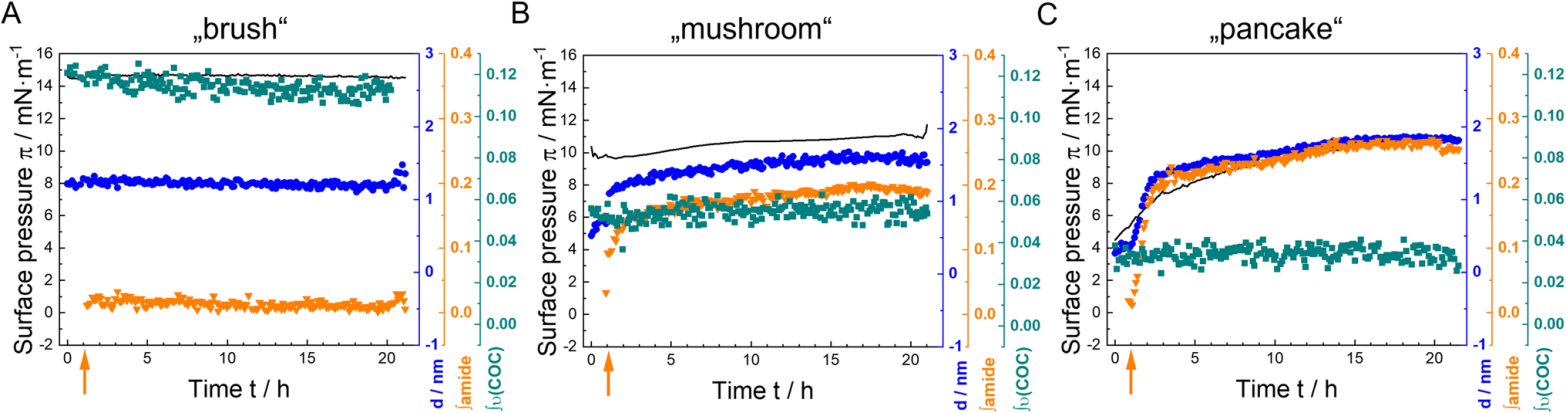
Competitive surface activity experiments of P188 and mAb, using simultaneous infrared reflection–absorption spectroscopy (IRRAS) and surface tension measurements. Different subphase concentrations, (**A** “brush” phase 1 mg/L, **B** “mushroom” phase 0.13 mg/L, **C** “pancake” phase 0.11 mg/L) were used. After one hour of adsorption time, mAb was injected (*c*_sub_ = 5 mg/L). Measurements were performed on 24.4 mM histidine buffer (pH = 6.0 ± 0.2) at a temperature of *T* = 20 °C. Surface pressure π (black line), layer thickness *d* (blue circles ●), integral of amide-I and amide-II-bands (green triangles ▼) and integral of asymmetrical C–O–C-stretching vibration centred at 1100 cm^−1^ (olive squares ■) as a function of time (h). The orange-coloured arrows show the injection time of mAb into the subphase (color figure online)

Experiments with selected P188 concentrations were performed with additional IRRAS detection. Figure 5 shows the results of three experiments with representative P188 concentrations, i.e., the time-dependent development of surface pressure π, layer thickness *d*, and the integrals of the amide bands as well as the integral of the asymmetrical C–O–C-stretching vibrational band. The related IRRA spectra are shown in Figure S6. The data indicate a comparable pattern to that observed in the previous studies employing simultaneous injection. A reduction in the P188 subphase concentration is associated with a decrease in the amount of adsorbed poloxamer and an increase in the amount of adsorbed mAb at the air–water interface. P188 existing in the densely packed “brush” phase (Fig. 5A) is able to completely prevent mAb adsorption. Here, the injection of mAb does not result in any observable change in the ν(C–O–C) integral, surface pressure, or layer thickness. All these measures remain at the values that were adopted by P188 “brush” phase formation, while the amide band integral is equal to zero. This shows that mAb is not able to reorganise or interact with pre-formed P188 brushes at the air–water interface.

To test the robustness of the surface shielding effect of a P188 “brush”, the experiment was repeated with a ninefold higher mAb concentration (*c*_sub_ = 45 mg/L). Despite this high mAb concentration in the subphase, no mAb was adsorbed at the interface, as it was effectively impeded by the P188 “brush” phase (Fig. S7).

The formation of a more loosely packed “pancake” phase prior to mAb injection resulted in a markedly different outcome. (Fig. 5C). In this case significant mAb adsorption takes place despite prior adsorption of P188. Concomitantly, the surface pressure increases from 4 mN∙m^-1^ to 10 mN∙m^−1^ and the layer thickness from 0.3 nm to 1.9 nm. However, mAb does not replace already adsorbed P188 from the interface, as can be deduced from the unaffected and constant integral of the P188 marker band. Consequently, the amount of adsorbed mAb (0.25 mRA/cm) is lower than in the case of simultaneous adsorption of P188 and mAb (0.35 mRA/cm). This shows that even in the “pancake” phase P188 has a partially protective effect on mAb adsorption.

Similar, but less pronounced effects can be observed when P188 was allowed to form a “mushroom*”* phase before injection of mAb (Fig. 5B and Fig. S6B). MAb is capable of adsorbing to the interface despite the presence of P188, while no P188 is displaced from the interface. The concentration of adsorbed mAb (0.2 mRA/cm) is lower than in the case of surface shielding by a “pancake” phase, but higher than in the case of simultaneous injection of P188 and mAb (0.18 mRA/cm). Interestingly, the surface pressure is only marginally influenced by mAb adsorption, showing that it is dominated by the more surface active P188.

**Fig. 6:**
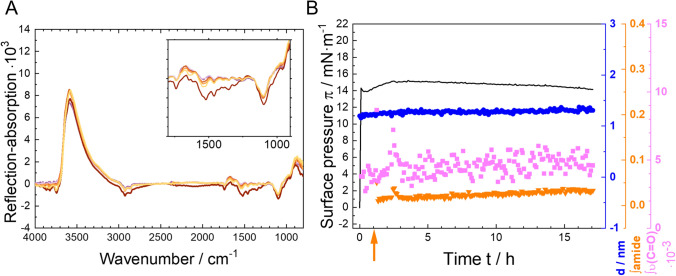
Competitive surface activity experiments of PS20 and mAb, using simultaneous Infrared reflection–absorption spectroscopy (IRRAS) and surface tension measurements. **A** PS20 subphase concentration of 1 mg/L was used. After about one hour adsorption time mAb was injected (*c*_sub_ = 5 mg/L). Measurements were performed on 24.4 mM histidine buffer (pH = 6.0 ± 0.2) at a temperature of *T* = 20 °C. A: IRRA spectra recorded 5 and 30 min after PS20 injection and at two to 15.5 h with additional mAb. The inset shows an enlarged part of the spectra in the wavenumber range of 1800 cm^−1^ to 900 cm^−1^. The color representation corresponds to different adsorption times. The purple graphs show the spectra of pure PS20. The spectra of PS20 and mAb are shown in orange. Darker shades of purple/orange indicate spectra recorded earlier, while lighter shades of purple/orange indicate spectra recorded later. **B**: Surface pressure π (black line), layer thickness *d* (blue circles ●), integral of amide I and amide II bands (green triangles ▼) and integral of C = O-stretching vibration centred at 1750 cm^−1^ (pink squares ■) as a function of time (h). The orange-coloured arrow shows the injection time of mAb into the subphase while PS20 was injected at *t* = 0 h (color figure online)

In addition, we tested the protective effect of a pre-formed PS20 layer (*c*_sub_ = 1 mg/L) on mAb adsorption (Fig. 6). The results are similar to those obtained for P188 in “brush” phase concentrations. The IRRA spectra in Fig. 6A show no amide bands and the respective integrals are roughly zero, showing that no mAb adsorption occurred when PS20 is present at the interface. Consequently, neither layer thickness nor surface pressure are affected by mAb injection into the subphase.

### Surfactant injection underneath a preformed antibody layer

**Fig. 7:**
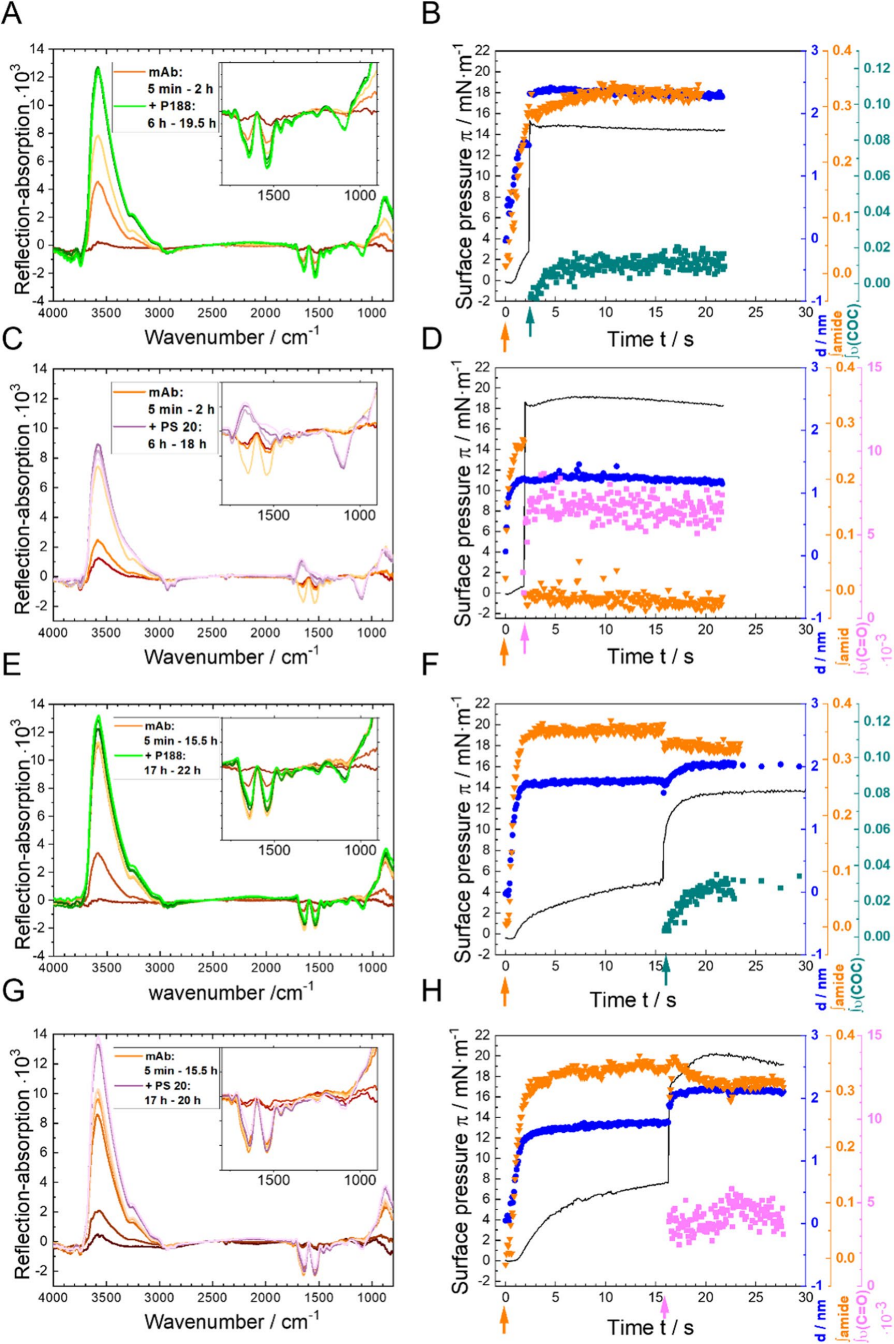
Competitive surface activity experiments of P188 (**A**, **B**, **E**, **F**), PS20 (**C**, **D**, **G**, **H**), and mAb, using simultaneous infrared reflection–absorption spectroscopy (IRRAS) and surface tension measurements. A mAb subphase concentration of 5 mg/L was used. MAb was injected at *t* = 0 h. After about two hours (**A**–**D**) or 17 h (**E**–**H**) of mAb film formation the surfactants were injected (*c*_sub_ = 1 mg/L). Measurements were performed on 24.4 mM histidine buffer (pH = 6.0 ± 0.2) at a temperature of *T* = 20 °C. **A**, **C**, **E**, **G** selected IRRA spectra at various times before and after mAb injection. The insets show enlarged parts of the spectra in a wavenumber range of 1800 cm^−1^ to 900 cm^−1^. **B**, **D**, **F**, **H** Surface pressure π (black line), layer thickness *d* (blue circles ●), integral of amide I and amide II bands (green triangles ▼) and integral of asymmetrical C–O–C stretching vibration centred at 1100 cm^−1^ (olive squares ■) or integral of C = O stretching vibration centred at 1750 cm^−1^ (pink squares ■) as a function of time. The orange-coloured arrows shows the injection time of mAb into the subphase, the green arrows shows the injection time of P188 into the subphase, the pink arrows shows the injection time of PS20 into the subphase (color figure online)

To investigate whether an already formed mAb layer can be desorbed from the interface by the injection of surfactants, we performed the experiments also in the inversed injection sequence, i.e., mAb was allowed to form an adsorption layer and subsequently, the surfactants were injected underneath the film, at two distinct time points after onset of mAb film formation. The results are summarised in Fig. 7.

In an initial experiment, P188 was injected two hours after the mAb injection, i.e. immediately after the adsorbed mAb film reaches its equilibrium layer thickness of approximately 1.5 nm and full mAb adsorption was confirmed (Fig. 7A, B). P188 was injected at a concentration that leads to the formation of a “brush*”* phase at a pure air–water interface (1 mg/L), and which had been demonstrated to inhibit the adsorption of mAb. In the now performed experiment, the P188 injection leads to an immediate and significant increase in surface pressure, from 3 to 15 mN∙m^−1^, accompanied by a notable expansion in layer thickness, from 1.5 to 2.3 nm. Additionally, the IRRA spectra exhibited the emergence of ν(COC) bands, which are indicative of evolving structural changes. These observations collectively indicate that P188 is adsorbed at the interface, despite the presence of mAb. Interestingly, also the amide band intensity further increases upon P188 injection. This demonstrates that P188 does not displace mAb from the interface, despite its higher surface activity. The rather unexpected increase in amide band intensity might result from secondary structure transitions and/or reorganisations in the already adsorbed protein layer. The amount of adsorbed P188 is low (0.01 mRA/cm). From the integral values it can be concluded that the average P188 surface coverage is only about 10 % of that of a P188 in a brush phase (0.11 mRA/cm), even though the surface pressure increases to typical brush phase values (compare to Fig. 5A). It can be assumed that P188 forms small patches of brushes that dominate the surface pressure and compresses the adsorbed protein in a manner that influences its conformation. The average layer thickness is, however, dominated by the mAb layer (2.2 nm).

A different outcome is observed when the same experiment is conducted with PS20 in place of P188. The injection of PS20 underneath a pre-formed mAb layer leads to immediate desorption of mAb from the interface. This is evidenced by the disappearance of the amide bands from the IRRA spectra (Fig. 7C) and the reduction in amide band intensity to zero (Fig. 7D). Conversely, the C=O stretching vibrational band intensity and the surface pressure increase due to the adsorption of PS20 at the interface. The surface activity of PS20 is about twice as high as that of P188. This may explain that PS20 is capable of fully desorbing a formed mAb layer from the air–water interface, in contrast to P188. This is also the case when the mAb layer is formed from a ninefold higher bulk concentration (see Figure S8). Despite the elevated surface pressure, interfacial mAb concentration, and mAb layer thickness observed in this instance, the injection of PS20 resulted in the complete desorption of the mAb film from the interface.

These two experiments were repeated with the following modification: the mAb film was allowed to mature for 17 h before the surfactants were injected underneath (Fig. 7E–H). When P188 is injected after 17 h (Fig. 7E, F), the film reacts similar to the case where the injection was already carried out after 2 h. That is, an increase in surface pressure to approximately 14 mN∙m^−1^, a slight increase in layer thickness from 1.8 to 2 nm, accompanied by the appearance of ν(COC) vibrational bands at 1100 cm^−1^, indicating the adsorption of P188 molecules at the interface, while the mAb is not desorbed. However, the amide band integral decreases slightly from approximately 0.35 mRA/cm to approximately 0.3 mRA/cm upon addition of P188. This might be interpreted as a minor part of the mAb being displaced from the surface. Altogether, P188 is not able to fully displace a mAb layer of 17 h from the air–water interface.

The injection of PS20 after 17 h beneath a matured mAb layer (Fig. 7G, H) results in a complete reversal of the observed phenomenon when compared to the injection after 2 h. While the surface pressure also increases to approximately 20 mN∙m^−1^, much less PS20 is adsorbed, as can be concluded from the lower integral intensities of the ν(C=O) band and the absence of a ν(COC) vibration centred at 1100 cm^−1^ in the spectra. Remarkably, only a minor fraction of mAb is displaced from the interface, while the major fraction resists the displacement by PS20. This is analogous to the injection of P188 after 17 h, but contrary to the injection of PS20 after 2 h, where the protein is completely desorbed from the interface. Apparently, the maturation of the mAb film increases its resistance to desorption from the interface.

With regard to this, it is notable that following the injection of mAb, the layer thickness as well as the amide band integral reach their equilibrium values after about three hours, while the surface pressure keeps increasing (Fig. 7F, H). This might be due to internal film reorganisation processes which lead to exposure of hydrophobic moieties of the protein without recruiting more material. Such reorganisation processes have already been previously described (Ortega-Vinuesa et al. 1998; Malmsten 1998).

The key question concerning the apparent maturation of the mAb layer is how this layer achieves its stability. A slow layer reorganisation may entail processes like protein unfolding and exposition of more hydrophobic molecular moieties to the air–water interface or the formation of protein intermolecular beta sheets. Similar processes have already been studied by Kanthe et al. (2021), who found that unfolding results in the exposure of hydrophobic amino acid residues such as isoleucine, leucine and valine, which are oriented towards the air-side of the surface (Kanthe et al. 2021). It has been previously postulated that monoclonal antibodies exist in a partially unfolded state at the air–water interface, with this denaturation facilitating the stabilisation of the molecule at the surface (Tronin et al. 1996). A detailed secondary structure analysis of IRRA amide bands is beyond the scope of this paper but will be the issue of further investigations and may help to explain the maturation process of the mAb layer at the air–water interface.

## Summary and conclusion

**Fig. 8:**
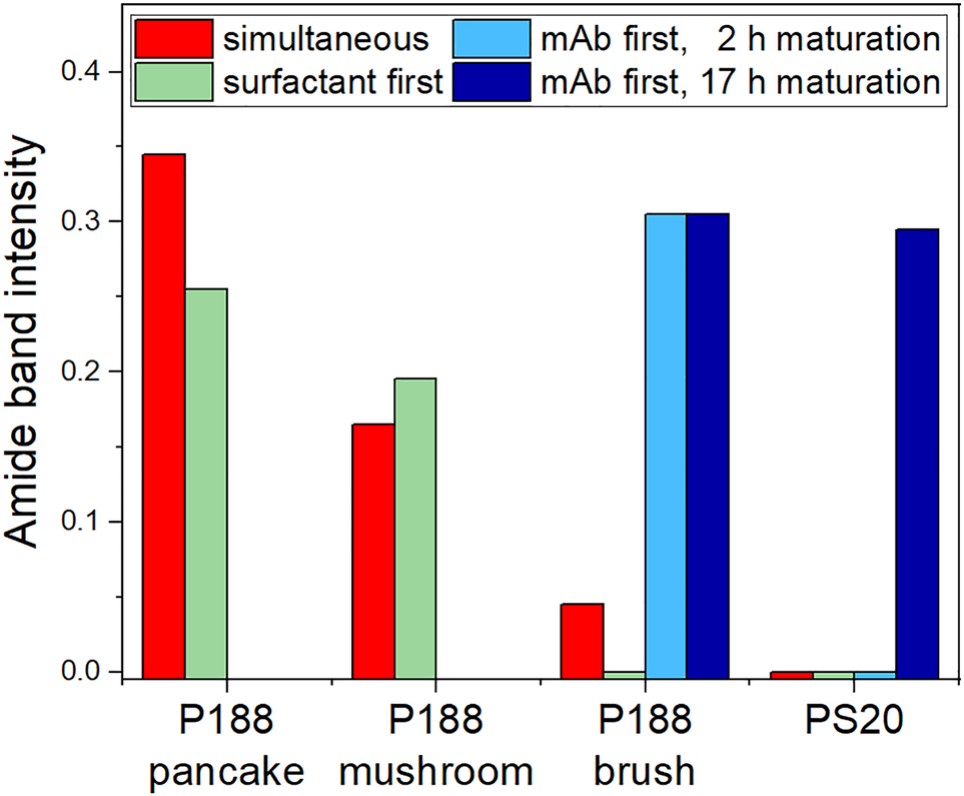
Integral amide I and II band intensity after the competitive adsorption of mAb and P188 or PS20 to the air–water interface. P188 was used in different concentrations (“pancake” phase 0.11 mg/L, “mushroom” phase 0.13 mg/L, “brush” phase 1 mg/L), whereas PS20 was always 1 mg/L. The mAb concentration was always 5 mg/L. The different bar colours denote the applied injection schemes. Red: simultaneous injection of mAb and surfactants, green: injection of mAb underneath a pre-formed surfactant layer, blue: injection of surfactants underneath a pre-formed mAb layer of 2 h (light blue) and of 17 h (dark blue) (color figure online)

**Scheme 4:**
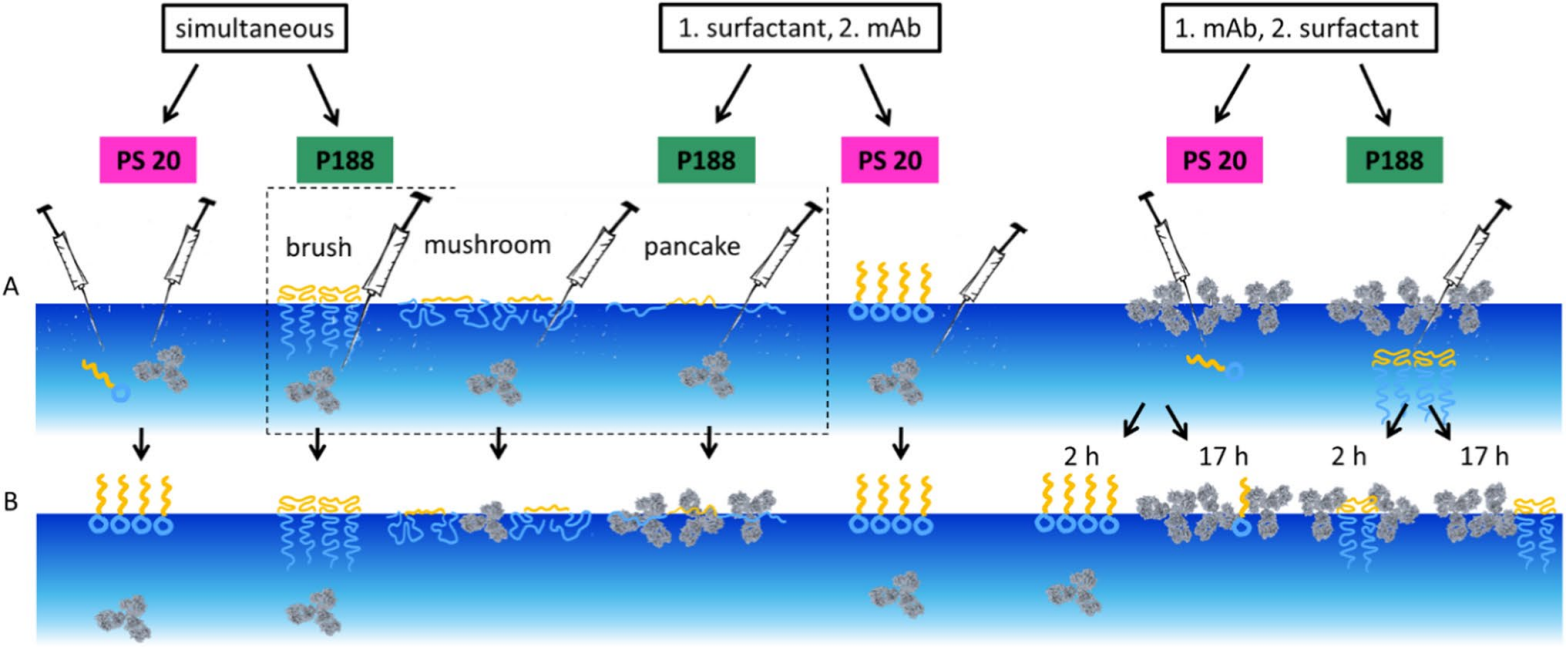
Concluding scheme. **A** Injection situation at the beginning of the measurement. **B** Results in competitive behaviour. Note: To enhance clarity regarding the phase states of P188, in **A** only the scheme for injecting mAb beneath a surfactant layer is shown. The schemes for simultaneous injection of mAb and the different P188-phase states are omitted. However, the results in **B** pertain to both experiments, simultaneous as well as prior P188 injection. For competitive experiments with an existing mAb layer, only the “brush” phase of P188 was used. Simultaneous injection of mAb and PS20 or P188 in the “brush” phase as well as mAb injection underneath a surfactant layer (PS20 or P188 in the “brush” phase) leads to a surfactant layer at the air–water interface, mAb remains in the subphase. Simultaneous injection of mAb and P188 in the “mushroom” phase as well as mAb injection underneath a P188 “mushroom” phase layer leads to a mixed layer of mAb and P188 at the air–water interface. Simultaneous injection of mAb and P188 in the “pancake” phase as well as mAb injection underneath a P188 “pancake” phase layer leads to a mAb layer at the air–water interface, P188 remains in the subphase. Surfactant injection underneath a mAb layer of two hours leads to a surfactant layer at the air–water interface with mAb remaining in the subphase in case of PS20. In case of P188, a mixed layer of mAb and P188 forms. Similar results are obtained with injection either PS20 or P188 underneath a 17-hour-old mAb layer (color figure online)

The principal findings of this study are summarized in Fig. 8, which provides a representation of the quantity of interfacially adsorbed mAb at different experimental conditions. The conditions in which mAb adsorption is prevented or reversed by the addition of surfactants can be clearly identified and correspond to the situation where the amide band intensity (bars in Fig. 8) are zero or close to zero. These are: the simultaneous or preceding addition of PS20 or P188 in “brush”-forming concentrations, and the posterior addition of PS20 when the mAb film age is < 2 h. In general, PS20 is more effective than P188 in preventing or reversing mAb adsorption to the air–water interface. Graphically, the different experimental conditions and injection schemes are summarized in Scheme 4.

In conclusion, the adsorption or replacement of the mAb at or from the air–water interface in the presence of a surfactant depend on several factors. These are:

i. the nature and amphiphilicity of the chosen surfactant,
ii. the surfactant subphase concentration,
iii. sequence of film formation, and
iv. the maturation time the mAb film.

The first point is evident, as PS20 is capable of completely shielding the surface and, in addition it is able to fully desorb a previously formed mAb layer up to two hours after its formation. P188 does not exhibit the latter effect in any of our performed experiments and the shielding is incomplete when simultaneous adsorption is permitted. In film balance measurements we, furthermore, demonstrated that the minimal concentration required to prevent mAb adsorption is significantly lower for PS20 (0.2 mg/L) than for P188 (1 mg/L) (see Figure S5). These effects correlate with the higher surface activity of PS20 in comparison to P188. We ranked the surface activities of the tested substances as follows: $\pi_{PS 20} > \pi_{P188} > \pi_{mAb}$. This suggests that the surface activity contributes to the efficacy of the surfactant in replacing the protein from the interface. However, this is not a sufficient criterion to assess whether a substance is replacing another from the interface, since otherwise also P188 should be able to replace mAb.

The surfactant subphase concentration becomes important as it might lead to the formation of different phase states and layer structures at the interface, as can be observed for P188. In this context, the most effective approach to preventing the adsorption of mAb to the air–water interface is to utilise P188 in concentrations where it undergoes self-assembly into the a “brush” phase. This notably corresponds to the P188 concentrations used in pharmaceutical drugs formulations. It was observed that P188 in the “pancake” phase cannot shield the surface and provide adequate protection for mAb adsorption, and that the mushroom phase only offered partial prevention of mAb adsorption. The concentration dependence is represented in Fig. 8, which depicts the reduction in the integral amide band intensity (red and green bars in Fig. 8) as P188 concentrations increase.

Furthermore, it could be shown that it is more straight forward to prevent the adsorption of mAb than to reverse the process once an interfacial mAb film has formed. The most effective method for preventing adsorption is to allow the surfactant to occupy the surface before adding the mAb to the subphase (green bars in Fig. 8). However, the simultaneous adsorption of mAb and surfactants yields comparable outcomes (red bars in Fig. 8), most likely due to the much faster adsorption kinetics of the surfactants as compared to mAb molecules (Fig. 2). Posterior injection leads to mAb desorption only for PS20 and could not be attained with P188 (blue bars in Fig. 8).

For desorption of a once formed mAb layer, the injection time of PS20 is decisive. It is imperative that PS20 is injected within the initial two hours following the injection of mAb, in order to ensure mAb displacement. A gradual and slow reorganisation of the mAb layer seems to occur over a time scale of several hours, which renders the matured mAb layer more resistant to desorption. Understanding the thermodynamic and structural aspects of these slow reorganisation processes within the mAb layer is highly intriguing avenue of research to optimise formulations.

## Supplementary Information

Below is the link to the electronic supplementary material.

Supplementary file1 (PDF 664 KB) Hingst_mAbadsorption_SI.pdf. They contain additional Figures, calculations of the critical micelle concentration (cmc), calculation of the area demand of mAb at the interface and images of the instruments.

Supplementary file2 (JPG 140 KB)

## Data Availability

All data supporting the findings of this study are available upon request.
