# Supplementary material for: Competitive adsorption of a monoclonal antibody and amphiphilic polymers to the air–water interface
Source: Eur Biophys J. 2025 May 22;54(5):213–29. doi: 10.1007/s00249-025-01752-0 (PMC12310791; doi:10.1007/s00249-025-01752-0)
Supplement: Supplementary file 1 — Supplementary file1 (PDF 664 KB) Hingst_mAbadsorption_SI.pdf. They contain additional Figures, calculations of the critical micelle concentration (cmc), calculation of the area demand of mAb at the interface and images of the instruments. [file 249_2025_1752_MOESM1_ESM.pdf]

SUPPORTING INFORMATION

COMPETITIVE ADSORPTION OF A MONOCLONAL ANTIBODY AND AMPHIPHILIC POLYMERS TO THE AIR-WATER INTERFACE

Elise J. Hingst[1], Michaela Blech[2], Dariush Hinderberger[1], Patrick Garidel[1,2], Christian Schwieger[1*]

[1] Institute of Chemistry, Physical Chemistry – Complex Self-Organizing Systems, Martin Luther University Halle-Wittenberg, Von-Danckelmann-Platz 4, 06120 Halle (Saale), Germany

[2] Boehringer Ingelheim Pharma GmbH & Co. KG. Innovation Unit, PDB-TIP, Birkendorfer Str. 65, 88397 Biberach (Riss), Germany

Content:

- **Instrument pictures**
- **Infrared Reflection-Adsorption Spectroscopy (IRRAS) band parameters**
- **Area demand of a monoclonal antibody (mAb) molecule and surface coverage**
- **Surface activity of pure compound by drop shape tensiometry**
- **Critical micelle concentration (*cmc*) calculations**
- **IRRA spectra of pure compounds after subtraction of H_2O contribution (to Fig 3, main text)**
- **IRRAS: simultaneous injection of mAb and polysorbate 20 (PS20)**
- **Film balance: mAb underneath surfactant film at various surfactant concentrations**
- **IRRA spectra to mAb injection after surfactant film formation (see Fig 5, main text)**
- **Experiments with increased mAb concentration (*c* = 45 mg/L)**
- **References**

Instruments pictures

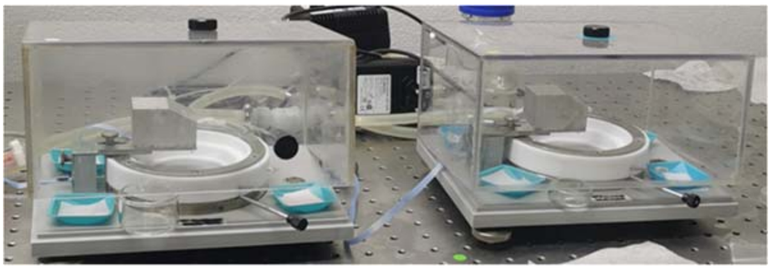

Scheme S1: Used Langmuir troughs for adsorption measurements.

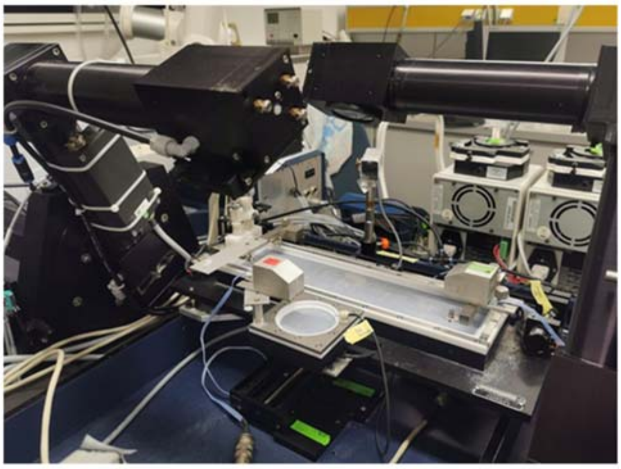

Scheme S2: Used IRRAS instrument.

IRRA band parameters

Table S1: Integration limits and –methods (OPUS software) for characteristic vibrations of selected functional groups. ν - stretching vibration, ν_a - asymmetric stretching vibration, δ - deformation vibration.

**Substance**	**Wavenumber / cm^{-1}**	**Classification**	**Functional group**	**Method**	**Integration limits / cm^{-1}**
**mAb**	amide I (1658)<br>amide II (1537)	ν (C=O)<br>ν (C-H)<br>δ (N-R_2)	amide	B	1485 - 1725
**P188**	1100	ν_a (C-O-C)	ether	F	1180 – 1050<br>baseline points:<br>1220; 1180; 1050; 980
**PS20**	1750	ν (C=O)	ester	B	1720 -1760

Area demand of a mAb molecule and surface coverage

For the assessment of the area $A_{molecule}$ that is occupied by a mAb molecule at the air-water interface, the molar volume V_m is used. According to calculations and experiments of Takashi Imai *et al.* (2005)[1], proteins with a molecular weight M of 146'000 g/mol have a molar volume of 102'460 cm³/mol. Based on a maximum layer thickness d of the pure antibody of 1.8 nm and according to the following calculation a value of the available area per molecule is obtained:

$$A_m = \frac{V_m}{d} = \frac{102460\ cm^3/mol}{1.8\ nm} = \frac{1.0246\times 10^{26}\ nm^3/mol}{1.8\ nm} = 5.692 \times 10^{25}\ nm^2/mol \qquad \text{(SI Eq.1)}$$

The area per molecule is determined by dividing the result of SI Eq.1 by the Avogadro constant which gives a molecular area of

$A_{molecule}$ = 94 nm² / molecule.

In comparison, the hydrodynamic diameter of mAb can be considered to be about 10 nm [2]. Under simplified assumption of a globular immunoglobulin, this gives a circular cross section area of around 79 nm². When assuming an anisometric mAb (Scheme 1), the occupied surface would be larger. Consequently, a mAb molecule would have an area requirement of at least 79 nm² at an available surface of about 94 nm² per molecule, which fits reasonably well with a literature value of 70 nm² [3]. It can therefore be concluded that the interface is almost entirely covered by the mAb.

Surface activity of the pure compounds determined by drop shape tensiometry

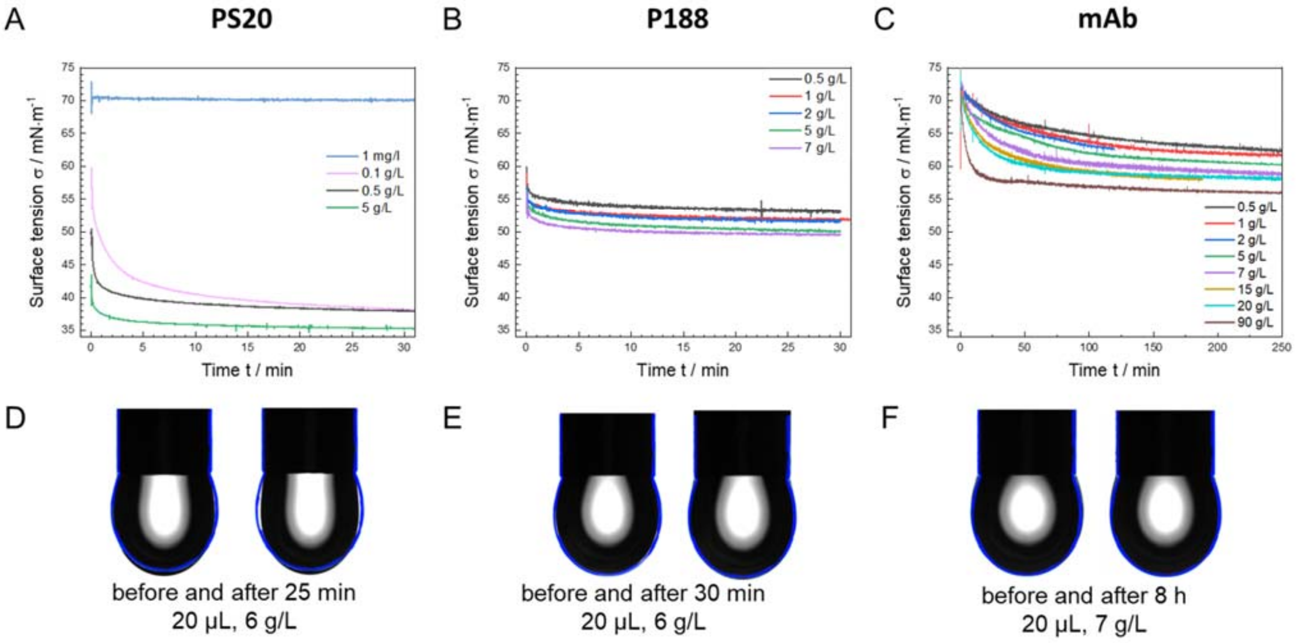

Figure S1: Surface behaviour of polysorbate 20 (PS20), poloxamer 188 (P188) and the monoclonal antibody (mAb) at different subphase concentrations. Measurements were performed in 24.4 mM histidine buffer (pH = 6.0 ± 0.2) at a temperature of T = 20 °C. A-C: Adsorption isotherms with the Drop Shape Tensiometer. D-F: Drop shapes compared to the shape of a water drop (blue outline).

cmc calculations

For the critical micelle concentration (*cmc*) range determination, the equilibrium surface tension was plotted as a function of the logarithm of the concentration and the cmc was determined from the intersection of two linear fits of the data (see SI Fig. 2). Literature values are shown on the x-axes [4]. For PS20, a value of 61 µM was experimentally determined, which agrees well with the literature value of 59 µM [5]. The experimentally determined value for P188 of 3.4 µM is over a factor of 100 lower than the literature value of 480 µM [4, 6], which also was the case in prior studies using film balance measurement data [7, 8]. In SI Fig. 2B a kink in the surface tension plots at 53 $mN{\cdot}m^{-1}$ becomes apparent that has also been found in previous experiments [6]. This behavior might be caused by the phase transition from "brush" to "cigar" which occurs in this region [9].

The concept of a *cmc* is used to describe self-assembly of amphiphilic molecules, in particular surfactants, and therefore does not apply to water soluble proteins like mAbs. Hence it is not surprising that the surface tension values for mAb were not in a state of equilibrium yet, even after 120 minutes, in contrast to the values for both surfactants, which were already taken after 20 minutes.

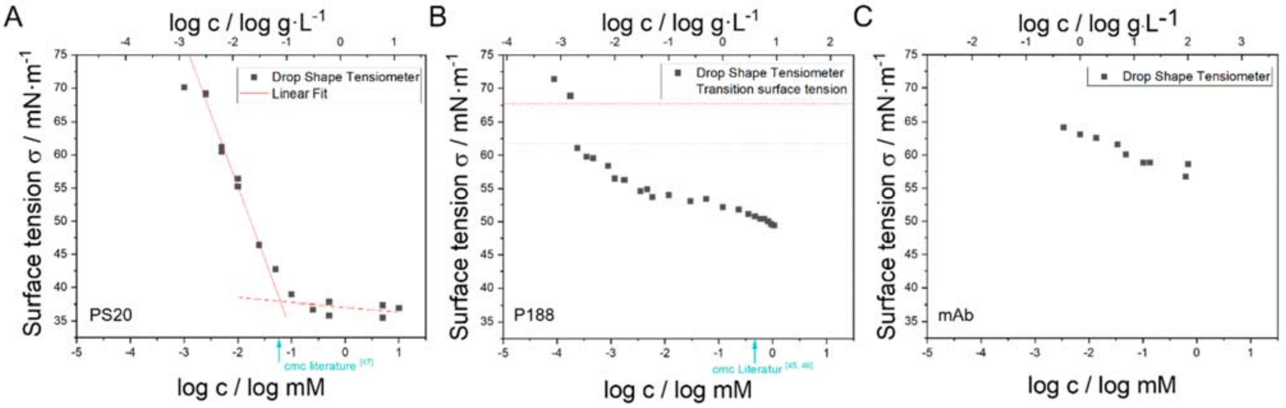

Figure S2: Equilibrium surface tensions as function of concentration of A: polysorbate 20 (PS20) after t = 20 min, B: poloxamer 188 (P188) after t = 20 min, and C: the monoclonal antibody (mAb) solution determined at *t* = 120 min. Measurements were performed in 24.4 mM histidine buffer (pH = 6.0 ± 0.2) at a temperature of *T* = 20 °C. Margins of error are within the size of the data points. In A liner fits for determination of the *cmc* are shown. The red dashed lines in B show the transition pressure of the "mushroom" phase to the "brush" phase at π = 11 $mN{\cdot}m^{-1}$, which is corresponds to σ = 61.8 $mN{\cdot}m^{-1}$ and of the "pancake" to "mushroom" phase at about π = 5 $mN{\cdot}m^{-1}$, which corresponds to σ = 67.8 $mN{\cdot}m^{-1}$.

IRRA spectra of Figure 3 (main text) after subtraction of H_2O contribution

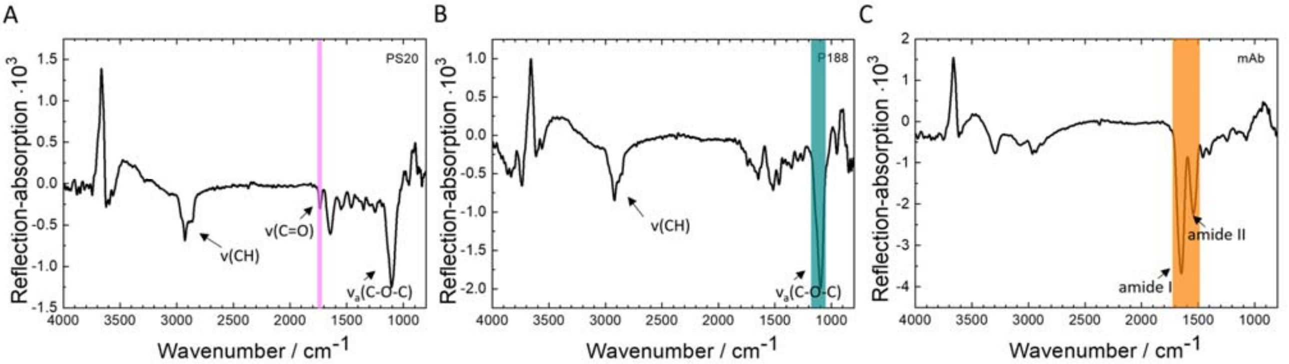

Figure S3: IRRA spectra of the pure compounds at the air-water interface after subtraction of simulated water spectra as used for further analysis. Measurements were performed on 24.4 mM histidine buffer (pH = 6.0 ± 0.2) at a temperature of *T* = 20 °C. A, B: after 30 minutes of adsorption, subphase concentration 1 mg/L; C: after 15 hours of adsorption, subphase concentration 5 mg/L. ν, stretching vibration; δ, deformation vibration. Integration limits of the respective bands are shown as coloured, transparent boxes.

IRRAS: simultaneous injection of mAb and PS20

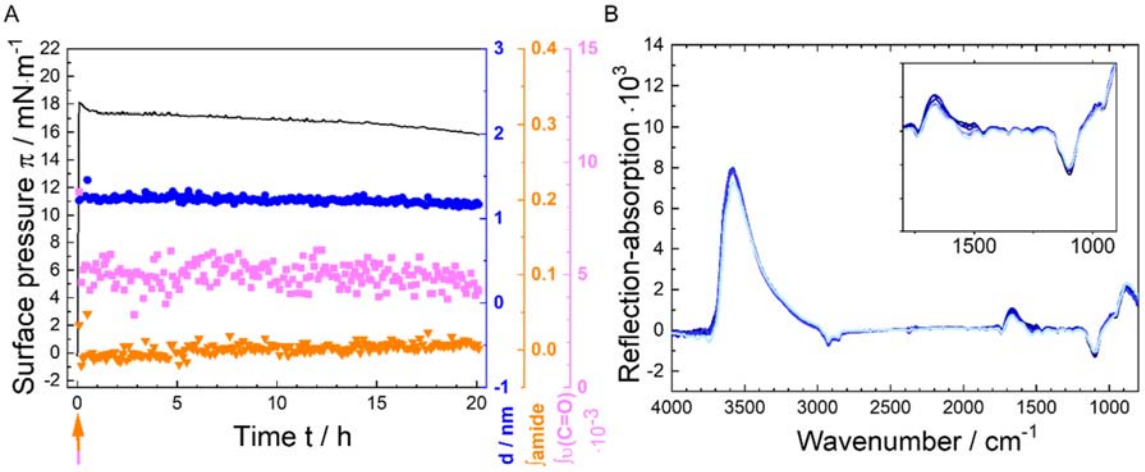

Figure S4: Competitive infrared reflection-absorption (IRRA) spectroscopic experiments of polysorbate 20 (PS20) and the monoclonal antibody (mAb). A simultaneous injection of mAb (c_{sub} = 5 mg/L) and PS20 (c_{sub} = 1 mg/L) was performed. Measurements were performed on 24. 4 mM histidine buffer (pH = 6.0 ± 0.2) at a temperature of T = 20 °C.
A: Surface pressure π (mN·m^{-1}) (black line), layer thickness d (●), integral of amide I and amide II bands (▼) and integral of C=O-stretching vibration centred at 1750 cm^{-1} (■) as a function of time / h. The pink-orange arrow indicates the injection time of PS20 and mAb into the subphase. B: IRRA spectra 5 minutes to 15.5 hours after PS20/mAb injection. The inset shows an enlarged part of the spectra in the wavenumber range of 1800 cm^{-1} to 900 cm^{-1}. The colour representation corresponds to different adsorption times. Darker shades of blue indicate spectra recorded at earlier time, while lighter shades of blue indicate spectra recorded at later time.

Film balance: mAb underneath the surfactant film at various surfactant concentrations

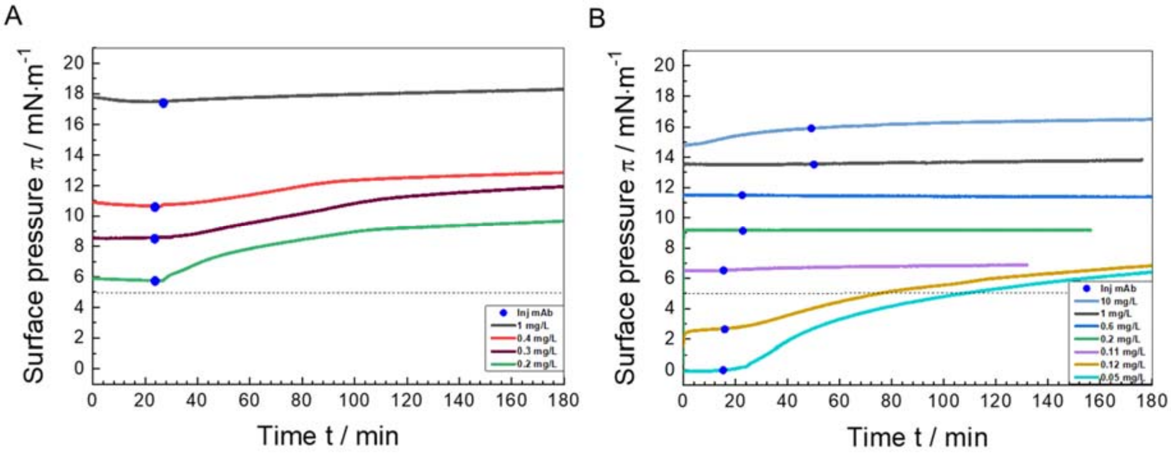

Figure S5: Competitive adsorption experiments using the Langmuir film balance of (A) poloxamer 188 (P188), (B) polysorbate 20 (PS20) and the monoclonal antibody (mAb). Surfactants were injected into the subphase at t = 0 h at different concentrations (see legends). Subsequently mAb was injected (c_{sub} = 5 mg/L) at the time indicated by blue circles (●). Measurements were performed on 24.4 mM histidine buffer (pH = 6.0 ± 0.2) at a temperature of T = 20 °C. The dotted black line shows the maximal attainable surface pressure of the mAb (c_{sub} = 5 mg/L) of about 5 mN·m^{-1}.

IRRA spectra of mAb injection after surfactant film formation (see Figure 5, main text)

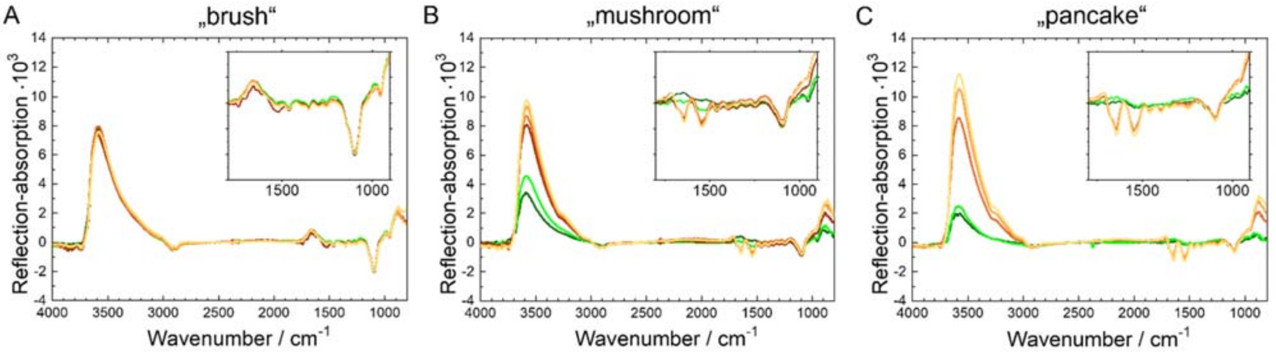

Figure S6: Competitive infrared reflection-absorption (IRRA) spectroscopic experiments of poloxamer 188 (P188) and the monoclonal antibody (mAb). Different subphase concentrations (A: "brush" phase, 1 mg/L; B: "mushroom" phase, 0.13 mg/L; C: "pancake" phase, 0.11 mg/L) were used. After one hour of adsorption time, the mAb was injected (c_{sub} = 5 mg/L). Measurements were performed on 24.4 mM histidine buffer (pH = 6.0 ± 0.2) at a temperature of T = 20 °C. A-C: IRRA spectra 5 to 30 minutes after P188 injection and two to 15.5 hours with additional mAb injection. The insets show an enlarged part of the spectra in the wavenumber range of 1800 cm^{-1} to 900 cm^{-1}. The colour representation corresponds to different adsorption times. The green graphs show the spectra of pure P188. The spectra of P188 and mAb are shown in orange. Darker shades of green/orange indicate spectra recorded earlier, while lighter shades of green/orange indicate spectra recorded later.

Experiments with increased mAb concentration (c = 45 mg/L)

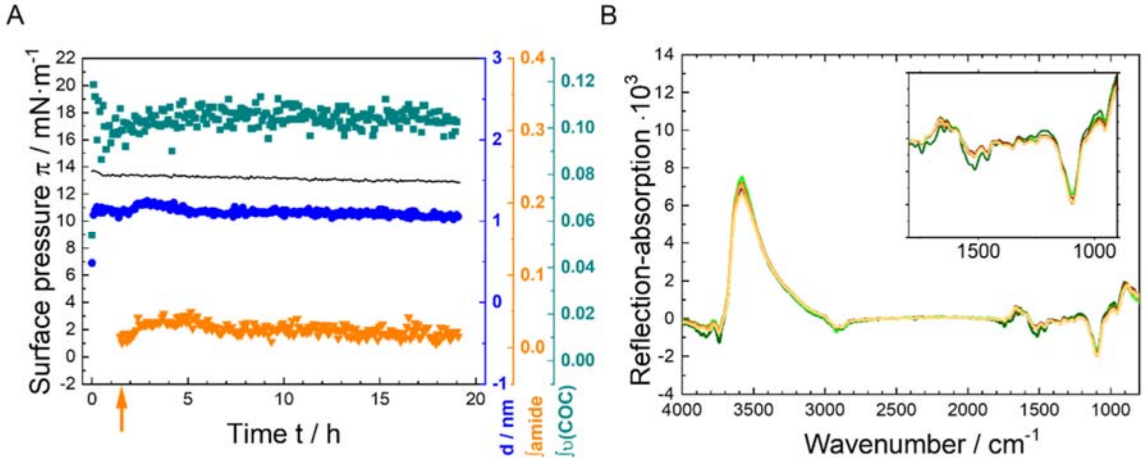

Figure S7: Competitive infrared reflection-absorption (IRRA) spectroscopic experiments of poloxamer 188 (P188) and the monoclonal antibody (mAb). A P188 subphase concentration of 1 mg/L was used leading to formation of a P188 "brush" phase. After about one hour adsorption time mAb was injected (c_{sub} = 45 mg/L). Measurements were performed on 24.4mM histidine buffer (pH = 6.0 ± 0.2) at a temperature of T = 20 °C. A: Surface pressure (black line), layer thickness d (●), integral of amide I and amide II bands (▼) and integral of asymmetrical C-O-C-stretching vibration centred at 1100 cm^{-1} (■) as a function of time. P188 was injected at t = 0 h into the subphase, the orange-coloured arrow shows the injection time of mAb into the subphase. B: IRRA spectra recorded 5 minutes to 30 minutes after P188 injection and at two to 15.5 hours after additional mAb injection. The inset shows an enlarged part of the spectra in the wavenumber range of 1800 cm^{-1} to 900 cm^{-1}. The colour representation corresponds to different adsorption times. The green graphs show the spectra of pure P188. The spectra of P188 and mAb are shown in orange. Darker shades of green/orange indicate spectra recorded earlier, while lighter shades of green/orange indicate spectra recorded later.

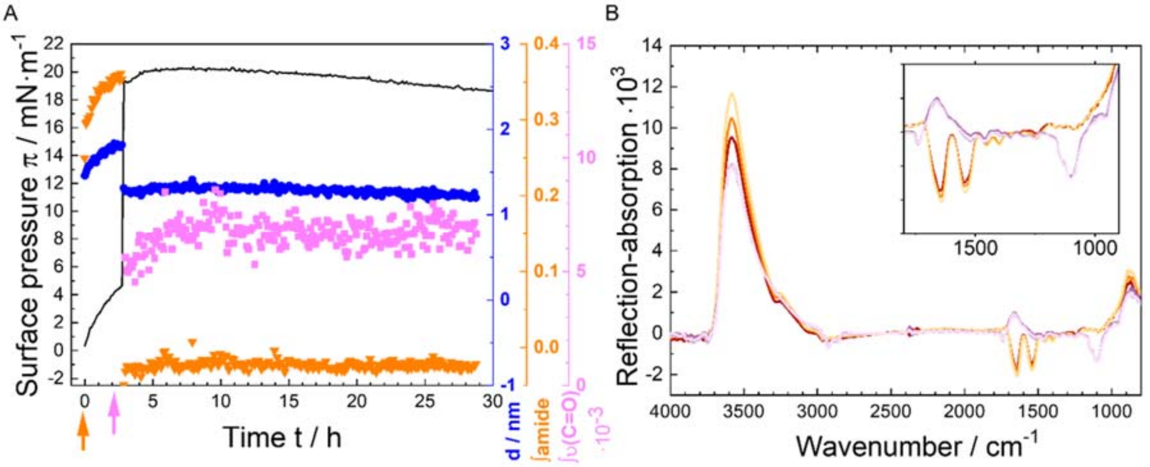

Figure S8: Competitive infrared reflection-absorption (IRRA) spectroscopic experiments of polysorbate 20 (PS20) and the monoclonal antibody (mAb). A mAb subphase concentration of 45 mg/L was used. After about two hours adsorption time, PS20 was injected (c_{sub} = 1 mg/L). Measurements were performed on 24.4 mM histidine buffer (pH = 6.0 ± 0.2) at a temperature of T = 20 °C. A: Surface pressure π (mN·m^{-1}) (black line), layer thickness d (●), integral of amide I and amide II bands (▼) and integral of C=O stretching vibration at 1750 cm^{-1} (■) as a function of time (h). The orange-coloured arrow and the pink arrow show the injection times of mAb and PS20 into the subphase, respectively. B: IRRA spectra recorded 5 minutes to two hours after mAb injection and 6 to 15.5 hours with additional PS20 injection. The inset in B shows an enlarged part of the spectra in the wavenumber range of 1800 cm^{-1} to 900 cm^{-1}. The colour representation corresponds to different time points. The spectra of pure mAb are shown in orange. The spectra of mAb and PS20 are shown in purple. Darker shades of orange/purple indicate spectra recorded earlier, while lighter shades of orange/purple indicate spectra recorded later.

References

(1) Imai, T.; Kovalenko, A.; Hirata, F. Partial molar volume of proteins studied by the three-dimensional reference interaction site model theory. *J Phys Chem B* **2005**, *109* (14), 6658-6665. DOI: 10.1021/jp045667c.

(2) Blech, M.; Horer, S.; Kuhn, A. B.; Kube, S.; Goddeke, H.; Kiefer, H.; Zang, Y.; Alber, Y.; Kast, S. M.; Westermann, M.; et al. Structure of a Therapeutic Full-Length Anti-NPRA IgG4 Antibody: Dissecting Conformational Diversity. *Biophys J* **2019**, *116* (9), 1637-1649. DOI: 10.1016/j.bpj.2019.03.036.

(3) Tronin, A.; Dubrovsky, T.; Nicolini, C. Comparative study of langmuir monolayers of immunoglobulin G formed at the air-water interface and covalently immobilized on solid supports. *Langmuir* **1995**, *11* (2), 385-389.

(4) Merck KGaA, D., Deutschland. *Kolliphor P 188*. 2023. https://www.sigmaaldrich.cn/CN/zh/product/sigma/15759 (accessed 17. December 2021).

(5) Knoch, H.; Ulbrich, M. H.; Mittag, J. J.; Buske, J.; Garidel, P.; Heerklotz, H. Complex Micellization Behavior of the Polysorbates Tween 20 and Tween 80. *Mol Pharm* **2021**, *18* (8), 3147-3157. DOI: 10.1021/acs.molpharmaceut.1c00406.

(6) Chunhachaichana, C.; Srichana, T. Efficiency of sildenafil encapsulation in poloxamer micelles. *Journal of Dispersion Science and Technology* **2019**, *40* (10), 1461-1468. DOI: 10.1080/01932691.2018.1518142.

(7) Powroznik, D. Master Thesis: Protein-Poloxamer-Wechselwirkungen an der Wasser-Luft-Grenzfläche. 2019.

(8) Bollenbach, L.; Trutschel, M.-L.; Gröger, S.; Garidel, P.; Mäder, K. Interfacial and self-association behaviour of poloxamer 188 in aqueous solutions. *J. Mol. Liq.* **2025**, *424*, 127119. DOI: 10.1016/j.molliq.2025.127119.

(9) Haefele, T.; Kita-Tokarczyk, K.; Meier, W. Phase behavior of mixed Langmuir monolayers from amphiphilic block copolymers and an antimicrobial peptide. *Langmuir* **2006**, *22* (3), 1164-1172. DOI: 10.1021/la0524216.
