# Supplementary figures and images for: Competitive adsorption of a monoclonal antibody and amphiphilic polymers to the air–water interface
Source: Eur Biophys J. 2025 May 22;54(5):213–29. doi: 10.1007/s00249-025-01752-0 (PMC12310791; doi:10.1007/s00249-025-01752-0)

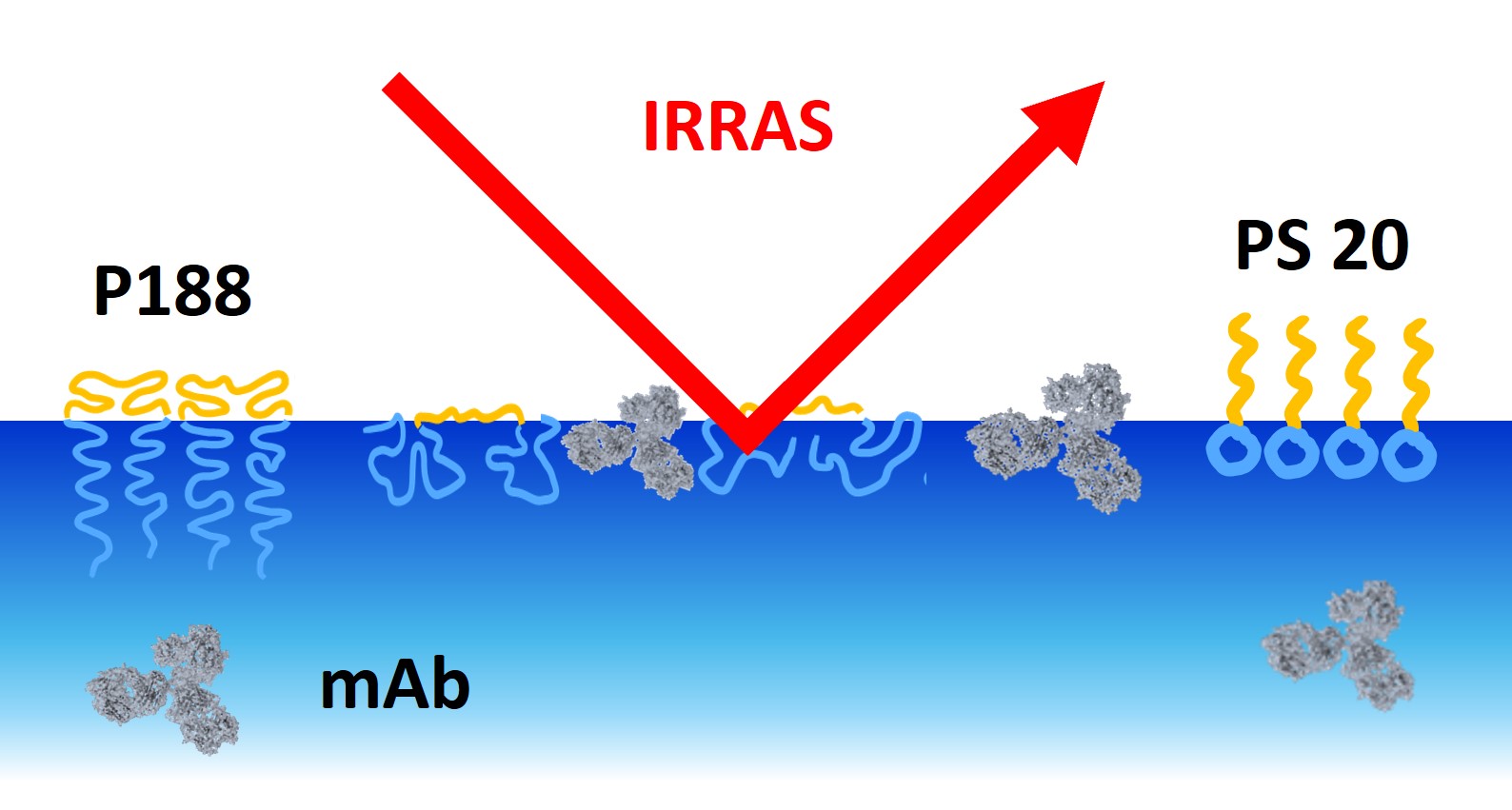

Supplement: Supplementary file 2 — Supplementary file2 (JPG 140 KB) [file 249_2025_1752_MOESM2_ESM.jpg]
